# Inhibition of angiogenesis by arsenic trioxide *via* TSP-1–TGF-β1-CTGF–VEGF functional module in rheumatoid arthritis

**DOI:** 10.18632/oncotarget.19867

**Published:** 2017-08-03

**Authors:** Juan Zhang, Chunling Li, Yining Zheng, Zhiguo Lin, Yue Zhang, Zhiyi Zhang

**Affiliations:** ^1^ Department of Rheumatology, The First Affiliated Hospital, Harbin Medical University, Nan Gang, Harbin, China

**Keywords:** angiogenesis, arsenic trioxide, rheumatoid arthritis, TGF-β1, VEGF, Pathology Section

## Abstract

Angiogenesis is a critical factor for rheumatoid arthritis (RA). Although anti-TNF biologics work effectively on some RA patients, concerns have been raised about the possible increased development of malignancies alongside such treatments. Arsenic trioxide (As_2_O_3_) has attracted worldwide attention and has been reported to treat some cancers. However, the effects of As_2_O_3_ on angiogenesis in the RA synovium remain unclear. Here, we report a systematic increased expression of TSP-1, TGF-β1, CTGF and VEGF in supernatants of a RA fibroblast-like synoviocytes (RA-FLS) and human dermal microvascular endothelial cells (HDMECs) co-culture compared with those from a normal human fibroblast-like synoviocytes (NH-FLS) and HDMECs co-culture. This increased expression may up-regulate endothelial tube formation and transwell migration, as well as microvessel sprouting in *ex vivo* aortic ring assay. These networked angiogenic factors mainly form a functional module regulating angiogenesis in the RA synovium. We show that As_2_O_3_ inhibits angiogenesis in the collagen-induced arthritis (CIA) synovium and consequently arthritis severity via significant suppression of TSP-1, TGF-β1, CTGF and VEGF expression in the CIA synovium, plus in the RA-FLS and HDMECs co-culture as well as NH-FLS and HDMECs co-culture system along with the presence or absence of TNF-α treatment. Thus As_2_O_3_ has a significant anti-angiogenesis effect on the RA-FLS and CIA synovium via its inhibition of the RA angiogenic functional module of TSP-1, TGF-β1, CTGF and VEGF and may have a potential for treating RA beyond cancer therapy.

## INTRODUCTION

Rheumatoid arthritis (RA) is characterized by pannus development. Angiogenesis is crucial for pannus development [1]. Fibroblast-like synoviocytes (FLS) in the synovium of RA may affect the vascular endothelium. The interaction between FLS and human dermal microvascular endothelial cells (HDMECs) via angiogenesis-related cytokines plays a crucial role in disease progression in RA. Connective tissue growth factor (CTGF) promotes the proliferation and migration of human umbilical vein endothelial cells (HUVEC) [2, 3]. TGF-β1 is a potent regulator of CTGF gene expression [4, 5]. The activation of latent TGF-β1 relies on thrombospondin-1 (TSP-1) binding to its precursor with latency-associated peptide (LAP) [6, 7]. On the other hand, it also has been reported that TSP-1 can be induced by TGF-β1 [8]. Furthermore, deregulated CTGF and TSP-1 are present in the synovial tissue of patients with RA [2] and CTGF, TGF-β1 and TSP-1 have been suggested to be possibly implicated in the pathophysiology of RA models [9, 10]. Vascular endothelial growth factor (VEGF) knockout mice showed reduced pathology and synovial angiogenesis in RA models [11]. However, the identification of functional modules is helpful for understanding the interdependence of gene to gene interactions in complex diseases such as RA as a whole, and the relationship among TSP-1, TGF-β1, CTGF and VEGF in RA pathophysiology is not fully characterized. A systematic study of TSP-1, TGF-β1, CTGF and VEGF, and their influence on neovascularization in RA is lacking but a must toward targeting them for clinical application.

Furthermore, we have shown that arsenic trioxide (As_2_O_3_, ATO) has an apoptotic effect on RA-FLS through the NF-κB signaling pathway in collagen-induced arthritis (CIA) and significantly improved arthritis in the rat model synovium. Pathological changes of the synovium were markedly relieved, so we speculated that As_2_O_3_ has a potential effect on anti-angiogenesis in RA [12]. As_2_O_3_ has attracted global attention because of its substantial anticancer activity in patients [13, 14] and murine solid tumors [15], and its derivative is considered as a novel orally-administrable angiogenesis inhibitor [16]. As_2_O_3_ prevented capillary tubule formation *in vitro* by inhibition of VEGF production in a leukemic cell line [17]. Similarly, it can inhibit solid tumor growth through suppressing VEGF expression [18]. Although anti-TNF biologics have been proven to be effective on some RA patients, concerns have been raised about the possible increased development of malignancies with such treatments. Therefore, we have quantitatively analyzed and identified the TSP-1, TGF-β1, CTGF and VEGF (TTCV) functional module in the angiogenesis in RA synovium and then investigated the effect of As_2_O_3_ on human fibroblast-like synoviocytes of RA patients and normal human with the presence or absence of tumor necrosis factor (TNF-α) treatment, as well as on microvessel sprouting in aortic ring assay *ex vivo* and CIA mice *in vivo*, exploring anti-angiogenesis molecular mechanisms of As_2_O_3_. In particular, we tested whether As_2_O_3_ has antirheumatic ability through inhibition of TTCV functional module-mediated angiogenesis.

## RESULTS

### Increased expression of TSP-1, TGF-β1, CTGF and VEGF in human RA-FLS

**Figure 1:**
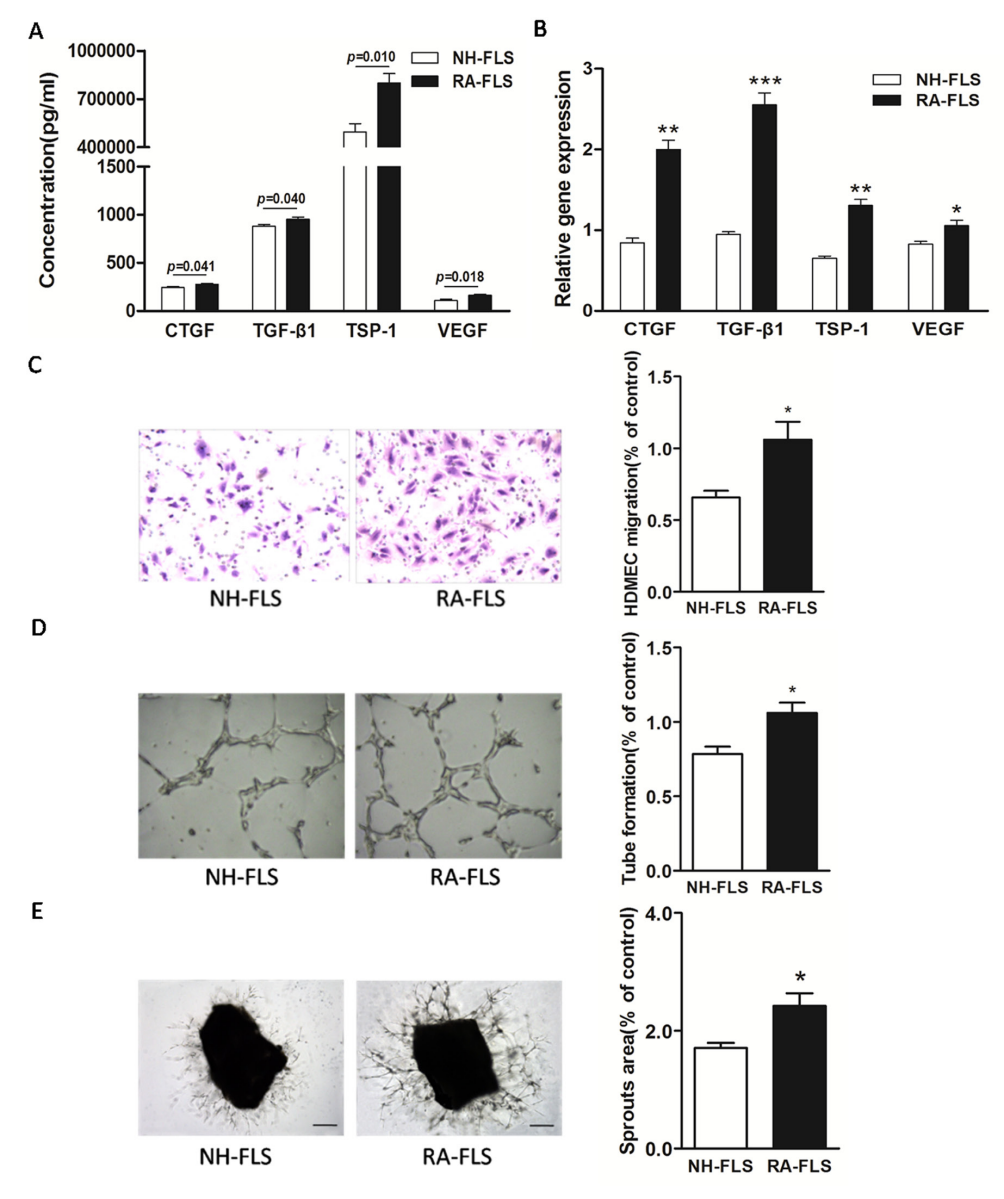
Increased expression of TSP-1, TGF-β1, CTGF and VEGF in supernatants of RA-FLS and HDMECs co-culture compared to NH-FLS and HDMECs co-culture Normal human (NH) FLS and rheumatoid arthritis (RA) FLS were co-cultured with HDMECs for 48 h, respectively. **A.** ELISA analysis demonstrated significant increase in the concentrations of TSP-1, TGF-β1, CTGF and VEGF in supernatants of RA-FLS and HDMECs co-culture (*n* = 3) compared with those from NH-FLS and HDMECs co-culture (*n* = 3; *p* < 0.05). **B.** Real-time PCR analysis showed increased mRNA expression of TSP-1, TGF-β1, CTGF and VEGF in RA-FLS co-cultured (*n* = 3) compared to NH-FLS co-cultured (*n* = 3; *p < 0.05, **p < 0.01, ***p < 0.001). **C.** and **D.** Transwell assay (C; *n* = 3) and tube formation test (D; *n* = 3) for 6 h demonstrated significant up-regulation in migration and capillary-like structure formation of HDMECs respectively under treatment of supernatants from RA-FLS and HDMECs co-culture (*n* = 3) compared to those from NH-FLS and HDMECs co-culture (*n* = 3; *p < 0.05). **E.** Mouse aortic rings were placed on GFR-Matrigel-coated plates and incubated in 1% FBS EGM-2. On 3^rd^ day, the EGM-2 were exchanged with supernatants from FLS and HDMECs co-cuture and further incubated for 3 days. *Ex vivo* aortic ring angiogenesis assay showed significant up-regulation in microvessel sprouting under treatment of supernatants from RA-FLS and HDMECs co-culture (*n* = 3) compared to those from NH-FLS and HDMECs co-culture (*n* = 3; *p < 0.05). Bars = 300 μm. Original magnification = ×5. Results are expressed as the mean ± S.E.M.

We first determined the expression of TSP-1, TGF-β1, CTGF and VEGF in human RA-FLS compared with FLS from normal human subjects (NH-FLS) using a FLS and HDMECs co-culture system. The ELISA analysis showed significantly enhanced expression of TSP-1, TGF-β1, CTGF and VEGF in supernatants of RA-FLS and HDMECs co-culture compared with those from NH-FLS and HDMECs co-culture (*p* < 0.05, Figure 1A). Similarly, we also observed a significant up-regulation in the mRNA expression of TSP-1, TGF-β1, CTGF and VEGF in RA-FLS from the lower chamber of the co-culture compared with NH-FLS, as quantified by real-time PCR analysis (*p* < 0.05, Figure 1B).

### Increased HDMECs migration, tube formation and microvessel sprouting were induced by co-cultured RA-FLS and HDMECs supernatants

Next, we asked if the increased expression of TSP-1, TGF-β1, CTGF and VEGF in human RA-FLS after secretion into the co-culture’s supernatants would promote HDMECs migration and tube formation. We conducted transwell analysis and tube formation test. Transwell assay and tube formation test demonstrated significant up-regulation in migration and capillary-like structure formation of HDMECs respectively under treatment of supernatants from NH-FLS and HDMECs co-culture as well as RA-FLS and HDMECs co-culture compared to those under management of unconditioned medium (*p* < 0.05, Supplementary Figure 1A-B). HDMECs migration induced by supernatants from co-cultured RA-FLS and HDMECs was significantly greater than that in supernatants from NH-FLS and HDMECs co-culture (*p* < 0.05, Figure 1C, Supplementary Figure 1A). Furthermore, we observed significantly enhanced vascularity, as determined by microvessel counts in Matrigel, in supernatants from co-cultured RA-FLS and HDMECs compared with those from co-cultured NH-FLS and HDMECs (*p* < 0.05, Figure 1D, Supplementary Figure 1B). In addition, mouse aortic rings *ex vivo* showed significant up-regulation in microvessel sprouting under treatment of supernatants from RA-FLS and HDMECs co-culture compared with those from co-cultured NH-FLS and HDMECs (*p* < 0.05, Figure 1E, Supplementary Figure 1C).

### TSP-1, TGF-β1, CTGF and VEGF stimulation and knockdown

**Figure 2:**
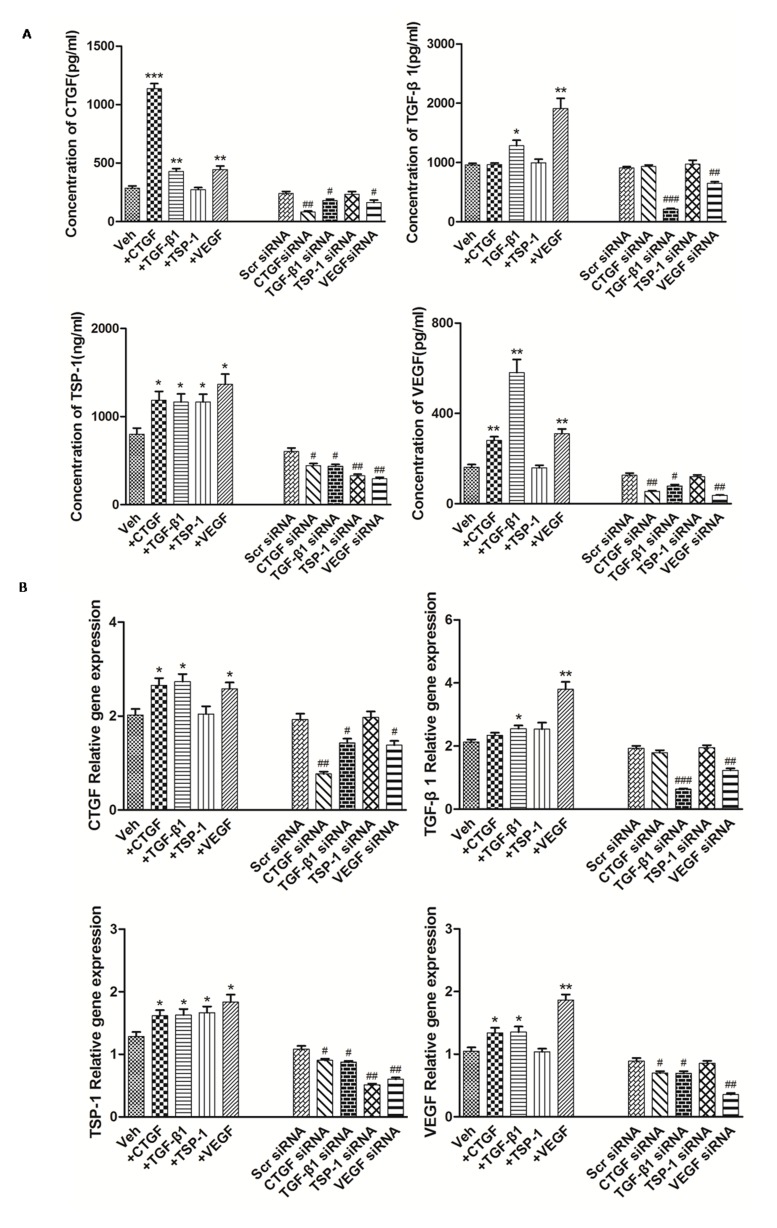
The modulation of TSP-1, TGF-β1, CTGF and VEGF expression alongside interventions of TSP-1, TGF-β1, CTGF and VEGF stimulation or knockdown **A.** The effects of TSP-1, TGF-β1, CTGF and VEGF stimulation/silencing in RA-FLS on TSP-1, TGF-β1, CTGF, and VEGF proteins expression in the supernatants were determined by ELISA. RA-FLS were stimulated with CTGF (500 ng/ml), TGF-β1 (5ng/ml), TSP-1 (1,000 ng/ml) and VEGF (50 ng/ml) respectively for 48 h. Stimulation (*n* = 3)/silencing (*n* = 3) of TGF-β1 in RA-FLS resulted in significant increase/decrease in the supernatants protein expression of TSP-1, CTGF, and VEGF (*p* < 0.05). Stimulation (*n* = 3)/silencing (*n* = 3) of VEGF in RA-FLS leaded to similar changes of the other three protein expression in supernatants (*p* < 0.05). Addition(n = 3)/silencing(*n* = 3) of CTGF in RA-FLS caused significant up-/down-regulation in the supernatants protein expression of TSP-1 and VEGF (*p* < 0.05). And stimulation (*n* = 3)/knockdown (*n* = 3) of TSP-1 in RA-FLS didn’t lead to significant increase/decrease in the expression of supernatants protein TGF-β1, CTGF and VEGF. **B.** TSP-1, TGF-β1, CTGF and VEGF mRNA expression levels were analyzed by real-time PCR after TSP-1, TGF-β1, CTGF and VEGF stimulation/silencing. Results showed similar alterations of TSP-1, TGF-β1, CTGF and VEGF mRNA expression as demonstrated in supernatants protein regulation after TSP-1, TGF-β1, CTGF and VEGF stimulation/silencing (*n* = 3 respectively; *p* < 0.05). Results are expressed as the mean ± S.E.M. *p < 0.05, **p < 0.01, ***p < 0.001 versus Veh, #*p* < 0.05, ##*p* < 0.01, ###*p* < 0.001 versus Scr siRNA. Veh = vehicle control. Scr siRNA = scramble siRNA.

Examination of differentially-expressed proteins showed that the factors TSP-1, TGF-β1, CTGF and VEGF were up-regulated in RA, but our understanding of their mutual regulatory network remains incomplete. To further clarify the relationships of TSP-1, TGF-β1, CTGF and VEGF regulation in RA-FLS, experiments with TSP-1, TGF-β1, CTGF and VEGF stimulation were carefully performed, followed by knockdown. The knockdown efficiency of TSP-1, TGF-β1, CTGF and VEGF was determined by real-time PCR and western blot analysis. At 48 h after transfection, 80% knockdown efficiency was achieved, as shown by both gene and protein expression (Figure 2B, Table 1B, Supplementary Figure 2). The results showed that the protein expression levels of CTGF, TSP-1 and VEGF were significantly up-regulated after TGF-β1 stimulation and significantly down-regulated after TGF-β1 knockdown (Figure 2A, Table 1A), whereas mRNA expression was significantly up-regulated after TGF-β1 stimulation, and significantly down-regulated after TGF-β1 knockdown (Figure 2B, Table 1B). Similar changes of TGF-β1, CTGF and TSP-1 mRNA and protein expression were observed after VEGF stimulation and knockdown (Figure 2, Table 1). Furthermore, the protein and mRNA expression levels of VEGF and TSP-1 were significantly up-regulated after CTGF stimulation and they were significantly down-regulated after knockdown of CTGF, whereas the expression of TGF-β1 did not change significantly compared with the controls (Figure 2, Table 1). In addition, the protein and mRNA expression of TGF-β1, CTGF and VEGF had no significant changes after TSP-1 stimulation and knockdown (Figure 2, Table 1).

Table 1The interdependent relationship of TSP-1, TGF-β1, CTGF and VEGF reflected by their expression levels in RA-FLS after interventions

**Table:** **A.** Concentration of TSP-1, TGF-β1, CTGF and VEGF proteins in the supernatants of RA-FLS culture after TSP-1, TGF-β1, CTGF and VEGF stimulation or knockdown.

Protein	+CTGF	+ TGF-β1	+ TSP-1	+ VEGF	CTGF siRNA	TGF-β1 siRNA	TSP-1 siRNA	VEGF siRNA
CTGF	3.99±0.14 ***	1.51±0.02 **	0.96±0.02	1.57±0.02 **	0.33±0.01 ##	0.75±0.02 #	0.98±0.02	0.68±0.01 #
TGF-β1	1.01±0.03	1.33±0.03 *	1.04±0.03	1.99±0.08 **	1.03±0.04	0.23±0.01 ###	1.07±0.04	0.72±0.01 ##
TSP-1	1.48±0.05 *	1.45±0.03 *	1.45±0.02 *	1.71±0.04 *	0.73±0.01 #	0.72±0.02 #	0.54±0.02 ##	0.48±0.01 ##
VEGF	1.74±0.07 **	3.61±0.20 **	0.99±0.03	1.94±0.05 **	0.42±0.01 ##	0.61±0.01 #	0.94±0.03	0.29±0.01 ##

Results are expressed as the mean ± S.E.M. Each intervention treatment group (n=3) was compared to its mock treatment control (*n* = 3). *p < 0.05, **p < 0.01, ***p < 0.001 versus vehicle control. # *p* < 0.05, ## *p* < 0.01, ### *p* < 0.001 versus scramble siRNA.

**Table:** **B.** TSP-1, TGF-β1, CTGF and VEGF mRNA expression levels of RA-FLS after TSP-1, TGF-β1, CTGF and VEGF stimulation or knockdown.

mRNA	+CTGF	+ TGF-β1	+ TSP-1	+ VEGF	CTGF siRNA	TGF-β1 siRNA	TSP-1 siRNA	VEGF siRNA
CTGF	1.32±0.00 *	1.35±0.00 *	1.01±0.00	1.27±0.00 *	0.40±0.00 ##	0.74±0.00 #	1.02±0.00	0.71±0.00 #
TGF-β1	1.10±0.00	1.20±0.00 *	1.14±0.00	1.79±0.00 **	0.93±0.00	0.33±0.00 ###	1.01±0.00	0.63±0.00 ##
TSP-1	1.26±0.00 *	1.27±0.00 *	1.30±0.00 *	1.43±0.00 *	0.83±0.00 #	0.74±0.00 #	0.49±0.00 ##	0.55±0.00 ##
VEGF	1.28±0.00 *	1.29±0.00 *	0.99±0.00	1.78±0.01 **	0.79±0.00 #	0.78±0.00 #	0.96±0.00	0.40±0.00 ##

Results are expressed as the mean ± S.E.M. Each intervention treatment group (*n* = 3) was compared to its mock treatment control (*n* = 3). *p < 0.05, **p < 0.01, ***p < 0.001 versus vehicle control. # *p* < 0.05, ## *p* < 0.01, ### *p* < 0.001 versus scramble siRNA.

### As_2_O_3_ treatment inhibited the up-regulated expression of the TSP-1, TGF-β1, CTGF and VEGF functional module in FLS and HDMECs co-culture system

To mimic the (strengthened) micro-environment of synovial tissue, we set up the NH-FLS and HDMECs co-culture as well as RA-FLS and HDMECs co-culture system respectively in transwell apparatus with the presence of TNF-α to mimic the inflammatory environment. We then investigated the effects of As_2_O_3_ on TSP-1, TGF-β1, CTGF and VEGF secretions in supernatants of each FLS and HDMECs co-cultures using ELISA.

**Figure 3:**
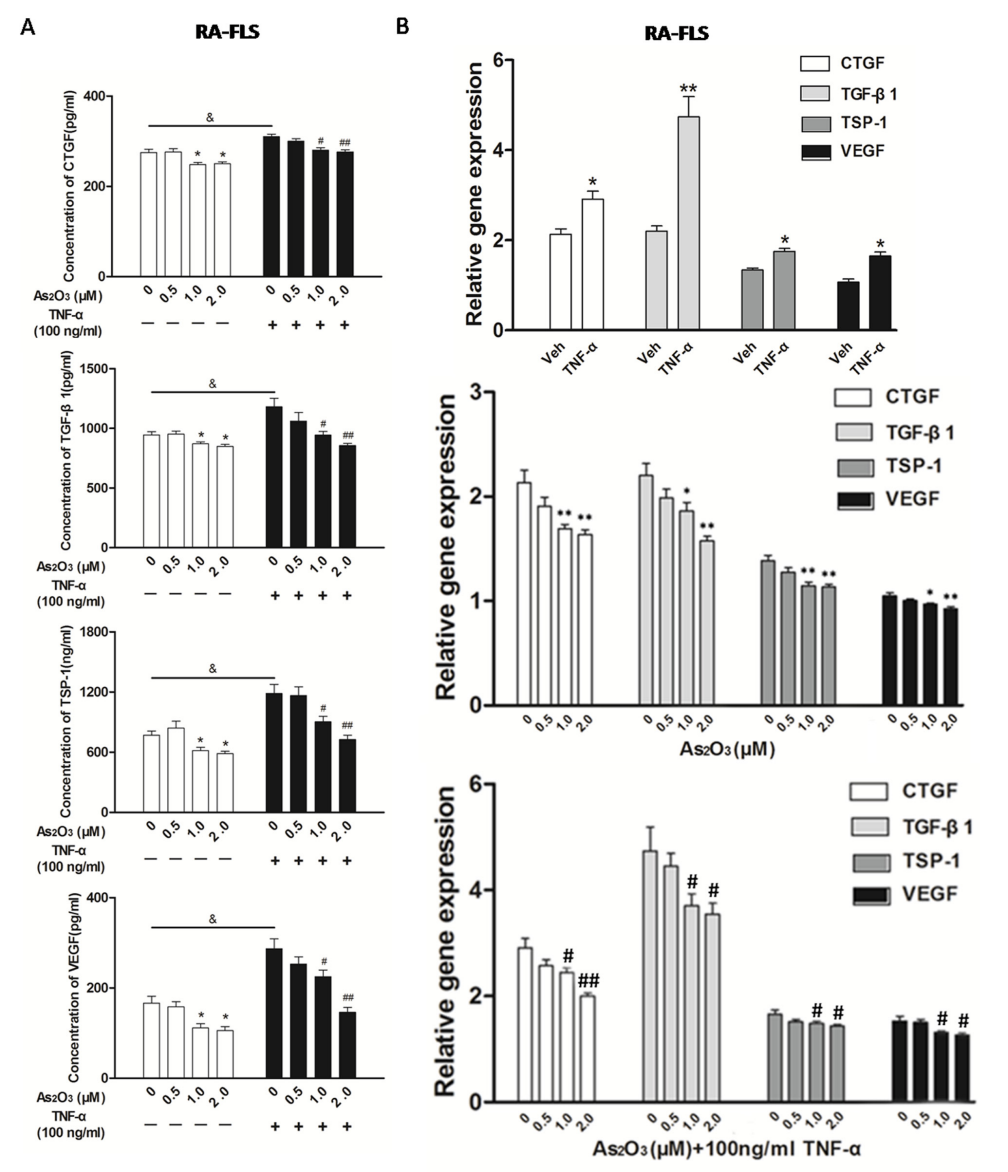

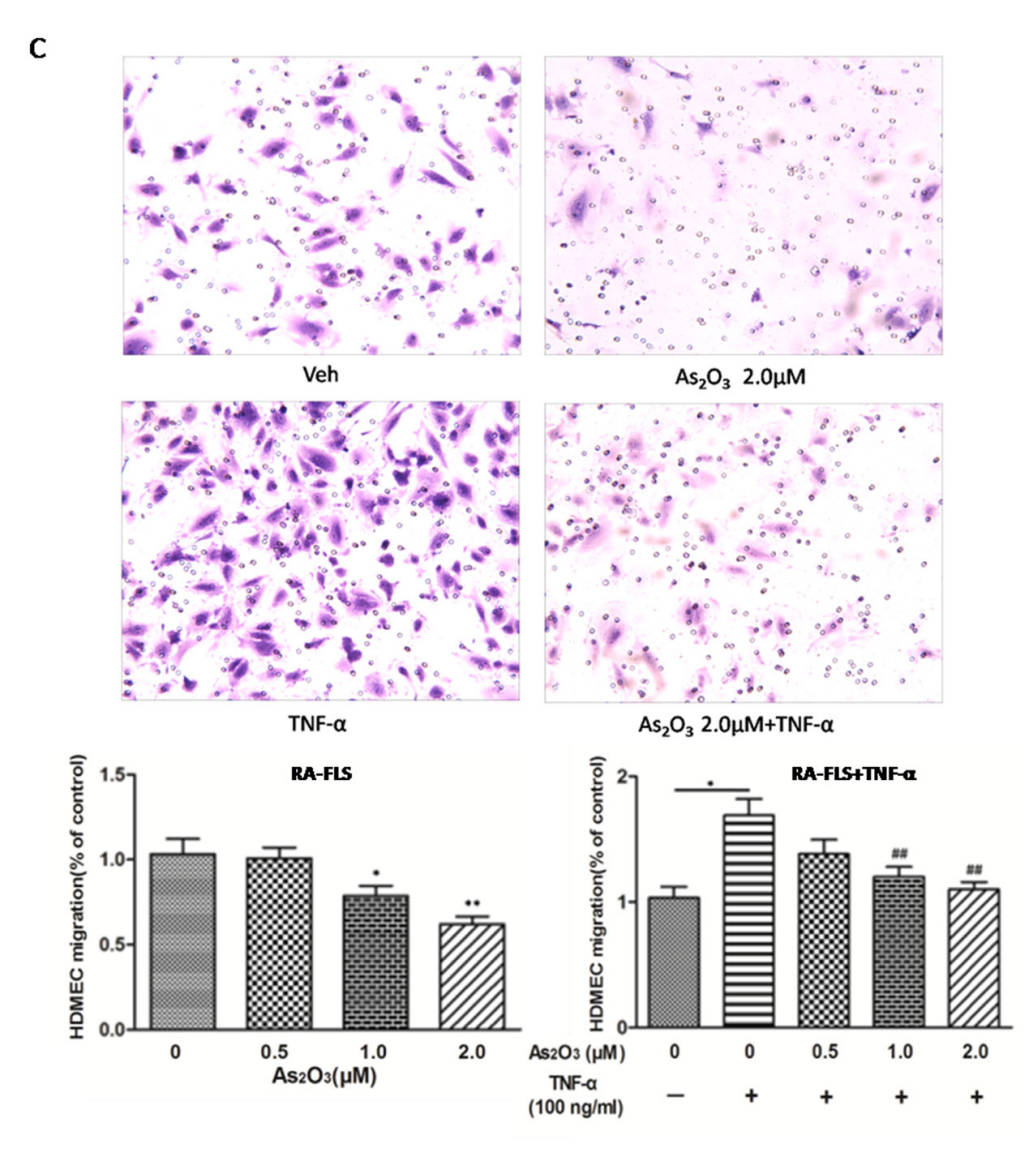

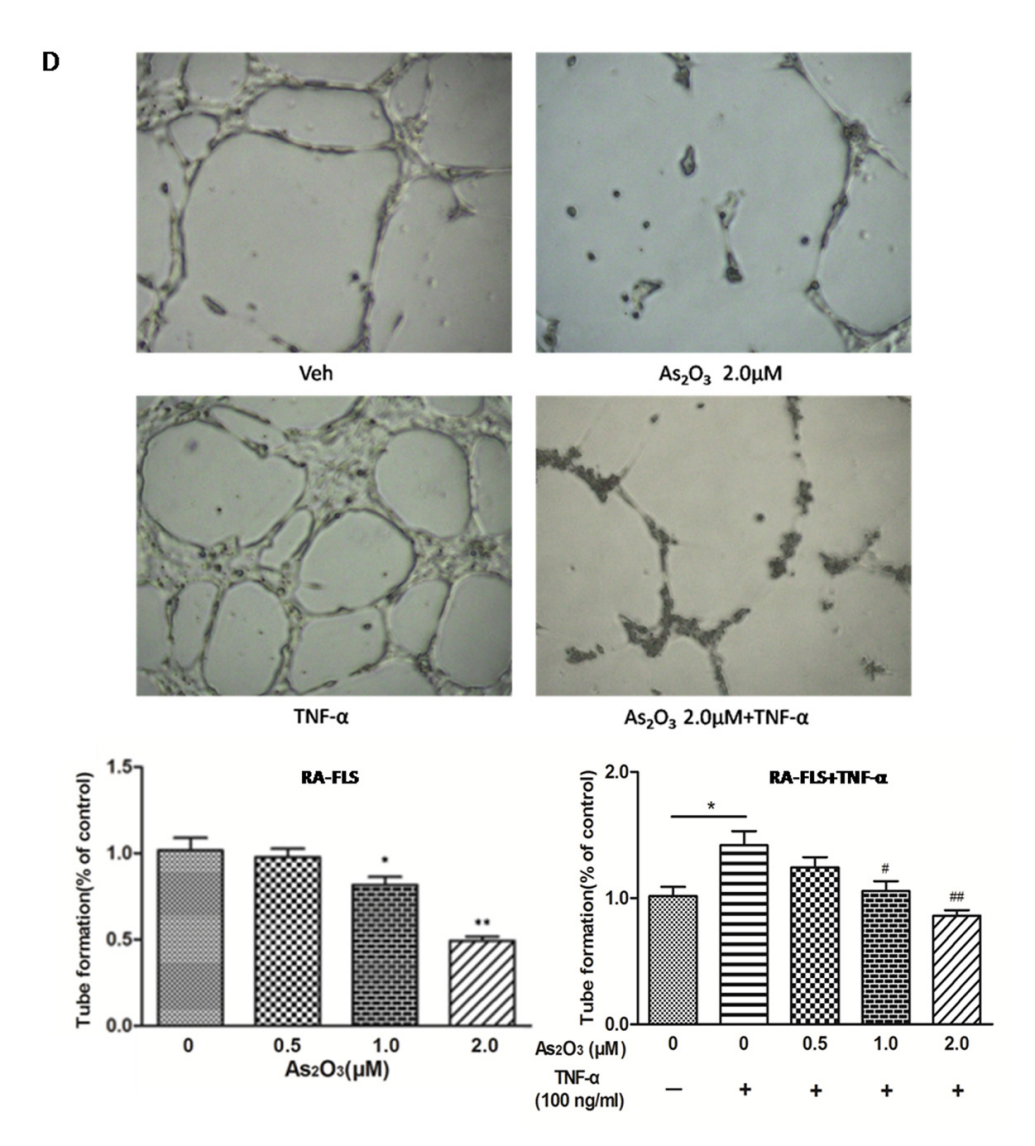

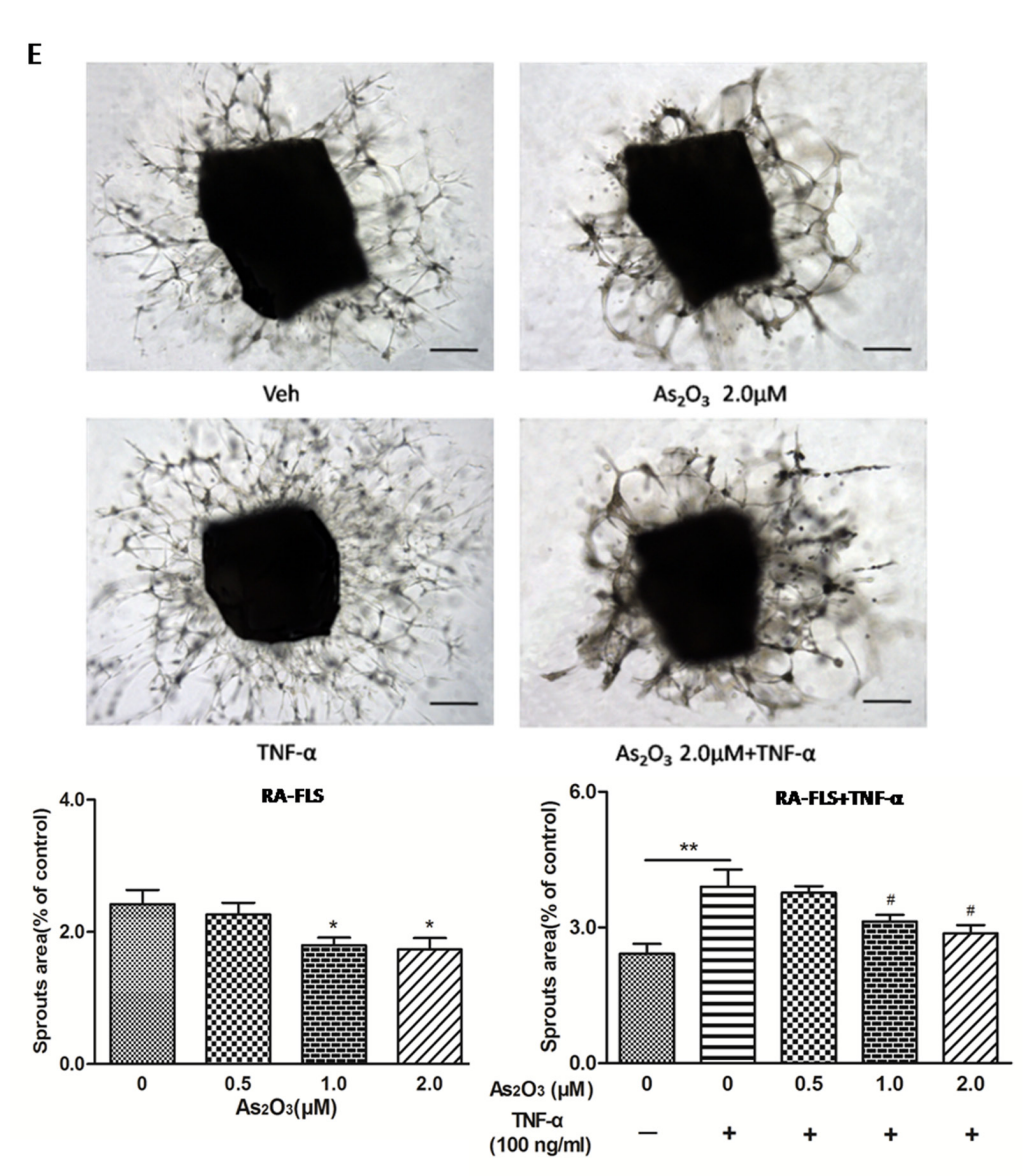
As_2_O_3_ inhibited angiogenesis by modulating TSP-1, TGF-β1, CTGF and VEGF expression in RA-FLS RA-FLS and HDMECs co-cultures were respectively treated with As_2_O_3_ alone or together with TNF-α (100ng/ml) for 48 h. **A.** TSP-1, TGF-β1, CTGF and VEGF protein expression in the supernatants from RA-FLS and HDMECs co-cultures were analyzed by ELISA. Results showed that concentrations of TSP-1, TGF-β1, CTGF and VEGF increased significantly after treatment with TNF-α (100 ng/ml) (*n* = 3) compared to vehicle control group (*n* = 3; & *p* < 0.05), and then significantly decreased concentrations of TSP-1, TGF-β1, CTGF and VEGF were observed after the treatment of As_2_O_3_ at doses of 1.0 μM and 2.0 μM (*n* = 3, respectively; #*p* < 0.05, ##*p* < 0.01). TSP-1, TGF-β1, CTGF and VEGF protein expression in the supernatants from RA-FLS and HDMECs co-cultures without TNF-α addition also decreased significantly after treatment of As_2_O_3_ at doses of 1.0 μM (*n* = 3) and 2.0 μM (*n* = 3) compared to vehicle control (*n* = 3; *p < 0.05). **B.** mRNA levels of TSP1, TGF-β1, CTGF and VEGF expression in RA-FLS co-cultured were performed by real-time PCR. Results showed similar changes of TSP-1, TGF-β1, CTGF and VEGF mRNA expression as demonstrated in protein regulation after treatment of As_2_O_3_ alone (*n* = 3) or together with TNF-α (100ng/ml) (*n* = 3; *p < 0.05, **p < 0.01, #*p* < 0.05, ##*p* < 0.01). **C.** and **D.** Transwell assay and tube formation test were performed by applying supernatants from RA-FLS and HDMECs co-culture to HDMECs for 6 h respectively. Results showed that migration and capillary-like structure formation of HDMECs significantly increased after TNF-α (100ng/ml) stimulation (*n* = 3, respectively; *p < 0.05), while As_2_O_3_ at doses of 1.0 μM (*n* = 3) and 2.0 μM (*n* = 3) played a significantly opposite role in migration and tube formation with or without TNF-α (*p < 0.05, **p < 0.01, #*p* < 0.05, ##*p* < 0.01), which were also in a dose dependent manner. **E.** *Ex vivo* aortic ring angiogenesis assay showed similar changes of microvessel sprouting as demonstrated in migration and tube formation of HDMECs in the transwell assay and tube formation test above (*n* = 3, respectively; *p < 0.05, **p < 0.01, #*p* < 0.05). Bars = 300μm. Original magnification = ×5. Results are expressed as the mean ± S.E.M. & *p* < 0.05 versus Veh. *p < 0.05, **p < 0.01 versus Veh, #*p* < 0.05, ##*p* < 0.01 versus TNF-α. Veh = vehicle control under treatment of 1% FBS DMEM alone. TNF-α = control group under treatment of TNF-α (100ng/ml).

The expression of TSP-1, TGF-β1, CTGF and VEGF proteins in the supernatants from NH-FLS and HDMECs co-culture as well as RA-FLS and HDMECs co-culture were significantly up-regulated by TNF-α induction (*p* < 0.05, Figure 3A and Supplementary Figure 4A), thereafter As_2_O_3_ significantly suppressed the secretion at a dosage of ≥1.0 μM in a dose-dependent manner (*p* < 0.05, Figure 3A and Supplementary Figure 4A). As_2_O_3_ could also down-regulated those four proteins in supernatants of RA-FLS and HDMECs co-culture when TNF-α treatment was absent (*p* < 0.05, Figure 3A), however those in supernatants from NH-FLS and HDMECs co-cultures under As_2_O_3_ administration without TNF-α treatment didn’t change significantly (Supplementary Figure 4A). Interestingly, the mRNA expression levels of TSP-1, TGF-β1, CTGF and VEGF in co-cultured FLS changed consistently with the protein expression levels (Figure 3B and Supplementary Figure 4B).

### As_2_O_3_ attenuates the angiogenesis *in vitro* and *ex vivo* systems

Since the expression of TSP-1, TGF-β1, CTGF and VEGF in the co-culture media could be suppressed by As_2_O_3_, we performed transwell analysis and tube formation assay to examine the effects of As_2_O_3_ on the angiogenesis of HDMECs with the supernatants from the co-cultures. First, we observed that HDMECs migration and tube formation were increased by supernatants from NH-FLS and HDMECs co-culture as well as RA-FLS and HDMECs co-culture under TNF-α treatment (*p* < 0.05, Figure 3C-3D, Supplementary Figure 3A-B, Supplementary Figure 5A-B). Thereafter the media with As_2_O_3_ addition significantly suppressed the HDMECs migration and tube formation at a dosage of ≥1.0 μM in a dose-dependent manner (*p* < 0.05, Figure 3C-3D, Supplementary Figure 3A-B, Supplementary Figure 5A-B). When TNF-α treatment was absent, the HDMECs migration and tube formation decreased significantly with management of supernatants from RA-FLS and HDMECs co-culture under treatment of As_2_O_3_ at doses of 1.0 μM and 2.0 μM compared to vehicle control group (*p* < 0.05, Figure 3C-3D, Supplementary Figure 3A-B). However, no significant down-regulation were seen under the application of the conditioned media from NH-FLS and HDMECs co-cultures with treatment of As_2_O_3_ (Supplementary Figure 5A-B). We also examined the effects of the conditioned media on microvessel sprouting using *ex vivo* aortic ring assay. Similar trends were obtained as those from the transwell analysis and tube formation assay (*p* < 0.05, Figure 3E, Supplementary Figure 3C, Supplementary Figure 5C).

### Effect of As_2_O_3_ on murine collagen II-induced arthritis

**Figure 4:**
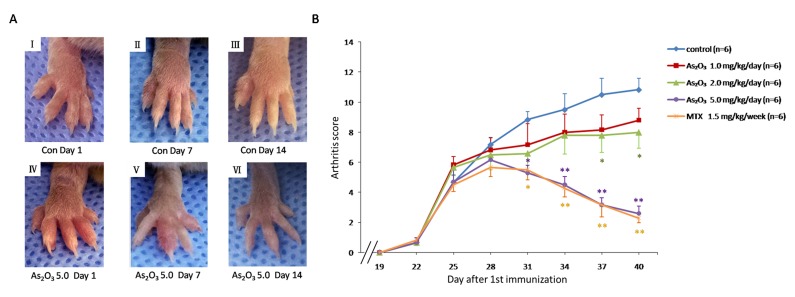
As_2_O_3_ prevented the progression of arthritis in CIA mice **A.** Representative photography of the hind limb from mice were shown. There was no obvious remission of arthritis in CIA control mice on the 1^st^(I), 7^th^(II) and 14^th^(III) day after drug treatment, while clinical symptoms of CIA mice treated with As_2_O_3_ at dose of 5.0 mg/kg/day had been improved significantly through the 1^st^(IV), 7^th^(V) and 14^th^(VI) days after drug administration. **B.** Arthritis scores of the mice were evaluated from 19^th^ day after primary immunization to 40^th^ day. Mice with CIA were administrated As_2_O_3_ and MTX intraperitoneally from 26^th^ day to 39^th^ day after 1^st^ immunization. The arthritis scores of normal group were nearly 0 on each test day (data not shown). CIA mice treated with As_2_O_3_ at doses of 2.0 mg/kg/day (*n* = 6), 5.0 mg/kg/day (*n* = 6) and MTX 1.5mg/kg/week (*n* = 6) showed significant improvement on arthritis score along with the treatment compared to the CIA control group (*n* = 6, *p < 0.05, **p < 0.01). Data are expressed as the mean ± S.E.M, Con = control = CIA control group. MTX = methotrexate.

To check if the anti-angiogenesis effect of As_2_O_3_ could be replicated *in vivo*, a murine collagen II-induced arthritis model was applied. The As_2_O_3_ treatment lasted from Day 26 to Day 39 after the first (primary) immunization. We assessed the severity of arthritis from Day 19 after primary immunization according to a standard arthritic score system. The mice with As_2_O_3_ treatment were classified at doses of 1.0, 2.0, or 5.0 mg/kg/day per mouse. CIA developed rapidly in paws after mice were immunized with Collagen II, and showed evident clinical symptoms (Figure 4A I, Figure 4A IV). Methotrexate (MTX) treatment (1.5 mg/kg/week), as a positive control, had a strong inhibitory effect on the development of arthritis (*p* < 0.05, Figure 4B). As_2_O_3_ at doses of 2.0 and 5.0 mg/kg/day exhibited continuously significant suppression in the arthritis scores from Day 26 to Day 40 (*p* < 0.05, Figure 4B), though the inhibition of arthritis in CIA mice was weak under the As_2_O_3_ treatment at a dose of 1.0 mg/kg/day. Representative pictures of the paws of mice from the CIA control group (Figure 4A I- III) and the 5.0 mg/kg/day As_2_O_3_ treatment group (Figure 4A IV-VI) on the 1^st^, 7^th^ and 14^th^ days after drug administration are shown. Mean body-weight of CIA control group decreased significantly compared to normal mice from Day 34 to Day 40. However, mean body-weight of groups under As_2_O_3_ and MTX treatment had no significant change compared to normal mice group (Supplementary Figure 6A). For better evaluation of As_2_O_3_ toxicity, the assessment for normal mice under different doses of As_2_O_3_ treatment were performed. Mean body-weight of each group of mice was calculated and used as a parameter of toxicity. The results showed that no significant body-weight loss was found among different treatments (as shown in Supplementary Figure 6B). In general, no other side effects, such as joint swelling, diarrhea, behavioral and hair abnormalities, or abnormal death, were observed during the study, indicating that As_2_O_3_ can suppress arthritis in CIA mice at non-toxic doses.

### As_2_O_3_ mitigated the severity of arthritis in CIA mice

**Figure 5:**
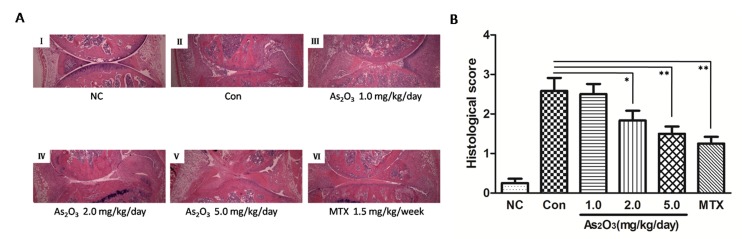
As_2_O_3_ inhibited histological damage in joints of CIA mice **A.** Hematoxylin and eosin staining of hind leg knee joint sections demonstrated no histological damage in normal control group (I), whereas synovial hyperplasia, pannus formation, massive joint destruction were observed in the knee joints of CIA control mice (II). CIA mice treated with MTX 1.5 mg/kg/week (VI) and As_2_O_3_ at doses of 1.0 mg/kg/day (III), 2.0 mg/kg/day (IV), and 5.0 mg/kg/day (V) demonstrated significantly diminished joint destruction, and this was particularly applied to CIA mice treated with MTX and As_2_O_3_ at doses of 2.0 and 5.0 mg/kg/day. Original magnification = ×4. **B.** Histological scores of CIA control mice (*n* = 6) increased significantly compared to normal control group (*n* = 6, *p* value not shown). CIA mice treated with MTX 1.5mg/kg/week (*n* = 6) and As_2_O_3_ at doses of 2.0 mg/kg/day (*n* = 6) and 5.0 mg/kg/day (*n* = 6) showed significant decrease in histological score compared to CIA control group (*n* = 6, *p < 0.05, **p < 0.01). Data are expressed as the mean ± S.E.M. NC = normal control mice. Con = CIA control mice. MTX = methotrexate.

To confirm the *in vivo* protection effects of As_2_O_3_, the severity of arthritis in CIA mice was further evaluated via histological analysis (hematoxylin and eosin stain, H&E staining) of knee joint sections. Synovial hyperplasia, pannus formation, massive joint destruction with erosion of cartilage and bone, and inflammatory cells with a high cumulative histological score were observed in the knee joints of CIA mice, whereas no synovial hyperplasia or pannus formation was found in normal mice (Figure 5A I-II, Figure 5B). However, consistent with the trend toward reduced paw swelling, there was significantly decreased inflammation, cartilage and bone destruction in mice in the As_2_O_3_ treatment group compared with mice in the phosphate buffered saline (PBS) treatment group (*p* < 0.05, Figure 5A II-V, Figure 5B).

After As_2_O_3_ treatment in CIA mice, joint destruction was significantly rescued in a dose-dependent manner. This was particularly evident in CIA mice after treatment with As_2_O_3_ at doses of 2.0 and 5.0 mg/kg/day (*p* < 0.05, Figure 5A III-V, Figure 5B). Indeed, the histological view of the synovial membrane in these groups was similar to the cases under MTX treatment (1.5 mg/kg/week) (Figure 5A VI, Figure 5B).

### As_2_O_3_ suppresses the expression of TSP-1, TGF-β1, CTGF and VEGF, and angiogenesis in the knee joints of CIA mice

**Figure 6:**
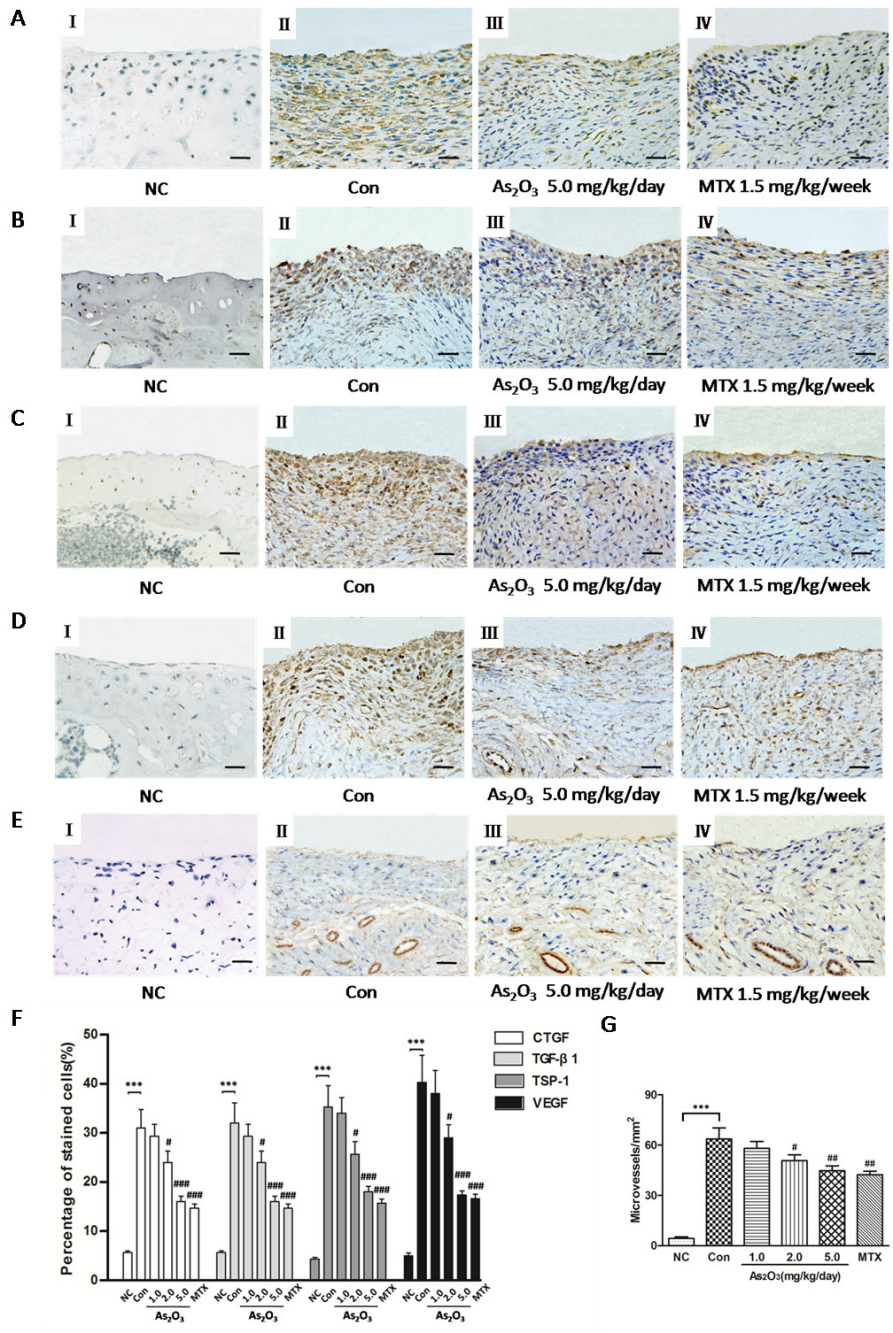
As_2_O_3_ suppressed TSP-1, TGF-β1, CTGF and VEGF expression and microvessel density in synovial tissue of CIA mice Positive staining appears as brown color. **A.**-**D.**, **F.** Immunohistochemical analysis demonstrated increased percentage (%) of positive cells for CTGF, TGF-β1, TSP-1 and VEGF in synovial tissue of CIA mice (*n* = 6; A-D, II, CIA control mice) compared to synovium of normal mice (*n* = 6; A-D, I, normal mice; F, ***p < 0.001), while CIA mice treated with As_2_O_3_ at a dose of 5.0 mg/kg/day (*n* = 6; A-D, III) and MTX 1.5mg/kg/week (*n* = 6; A-D, IV) showed decreased % of positive cells for CTGF, TGF-β1, TSP-1 and VEGF in synovial tissue compared to CIA control mice (*n* = 6; A-D, II; F, ###*p* < 0.001). Original magnification = ×40. Bars = 25μm. **E.**, **G.** Immunohistochemical analysis for vWF showed a significant increase in number of microvessels in synovial tissue of CIA control mice (*n* = 6; E, II) compared to the synovium of normal mice (*n* = 6; E, I; G, ***p < 0.001), while CIA mice treated with As_2_O_3_ at a dose of 5.0 mg/kg/day (*n* = 6; E, III) and MTX 1.5 mg/kg/week (*n* = 6; E, IV) demonstrated decreased number of microvessels in synovial tissue compared to CIA control mice (*n* = 6; E, II; G, ##*p* < 0.01). Original magnification = ×40. Bars = 25μm. Data are expressed as the mean ± S.E.M. ***p < 0.001 versus NC, #*p* < 0.05, ##*p* < 0.01, ###*p* < 0.001 versus Con. NC = normal control mice. Con = CIA control mice. MTX = methotrexate.

In order to understand effects of As_2_O_3_ on the expression of TSP-1, TGF-β1, CTGF and VEGF, and subsequently angiogenesis in the knee joints of CIA mice, we performed an immunohistochemical analysis and observed that normal mice showed weak or no immunostaining for TSP-1, TGF-β1, CTGF and VEGF (Figure 6A-6D I). However, CIA mice under PBS treatment had significantly increased staining intensity for TSP-1, TGF-β1, CTGF and VEGF in knee joint synovium (Figure 6A-6D II) compared with normal mice. Moreover, we saw significantly reduced staining intensity for TSP-1, TGF-β1, CTGF and VEGF in mice treated with As_2_O_3_ compared with CIA mice treated with PBS. Interestingly, immunostaining for TSP-1, TGF-β1, CTGF and VEGF were rare or weak under As_2_O_3_ treatment at doses of 2.0 and 5.0 mg/kg/day, similar to the effects of MTX (1.5 mg/kg/week)(Figure 6A-6D, III-IV). We calculated percentage (%) of positive cells for CTGF, TGF-β1, TSP-1 and VEGF in synovial tissue of mice and observed similar trends indicated in representative photography of the immunostained synovium (Figure 6F).

Finally, angiogenesis in the synovial tissue of CIA mice was measured via microvessel density (MVD) in the joint sections via von Willebrand factor (vWF) immunostaining. As_2_O_3_-associated anti-angiogenesis effects were further confirmed by measuring MVD. The immunohistochemical analysis showed that MVD was significantly stronger in the synovial tissue of CIA mice than in normal mice (*p* < 0.05, Figure 6E I-II, Figure 6G), and MVD decreased significantly under As_2_O_3_ treatment at doses of 2.0 and 5.0 mg/kg/day and MTX (*p* < 0.05, Figure 6E III-IV, Figure 6G). These results indicate that As_2_O_3_ may have protective effects through suppression of the TSP-1-TGF-β1-CTGF-VEGF functional module, subsequently having an anti-angiogenesis effect in the synovial tissues.

## DISCUSSION

RA is characterized by pannus formation, with extensive angiogenesis restricted to the synovium [1]. However, it is uncertain as to what kind of functional modules and which mechanisms may contribute to angiogenesis in the RA synovium. TSP-1, TGF-β1 and CTGF have previously been reported to be involved in the pro-inflammatory state in RA patients. In this study, we found that TSP-1, TGF-β1, CTGF and VEGF, as four angiogenic factors in the supernatants of the RA-FLS and HDMECs co-culture, were systematically up-regulated compared with those in the NH-FLS and HDMECs co-culture medium. These findings were in accordance with previous studies [9-11]. Importantly, these enhanced angiogenic factors may be functional, with increased vascularity, as demonstrated by transwell migration, tube formation and aortic ring assay.

In order to quantify the mutual interdependent regulatory interactions among these proteins in detail, we examined their responding expression levels by interventions using either stimulation or knockdown *in vitro*. We observed that expression of TSP-1, CTGF and VEGF may be up- or down-regulated by means of TGF-β1 stimulation or knockdown, respectively. TSP-1 and VEGF underwent similar changes after CTGF intervention. These results were consistent with earlier observations in different cell models [5, 8, 19, 20], and confirmed that CTGF was the downstream mediator of the effects of TGF-β1.

In addition, CTGF siRNA treatment has not significantly down-regulated TGF-β1 expression, although CTGF gene silencing was previously reported to prevent up-regulation of TGF-β1 gene expression in the liver after N-nitrosodimethylamine (NDMA) administration [21]. This raises our concerns for further assessing As_2_O_3_ potential tissue-specific effects in different tissues in near future so as to obtain the right dosage for clinical administration. Furthermore, we found that the expression of VEGF may have been increased by stimulation of TGF-β1 and CTGF, but decreased by their knockdown. Interestingly, the expression of TSP-1, TGF-β1 and CTGF could be up-regulated by VEGF addition and down-regulated by VEGF knockdown. Therefore, VEGF is the functional cytokine of the TTCV functional module in angiogenesis in RA synovial tissue. Lastly, CTGF also increases VEGF-dependent angiogenesis in human synovial fibroblasts [19].

Surprisingly, the expression of TGF-β1, CTGF and VEGF showed no significant change with TSP-1 intervention. These results are consistent with the results of Suzuki *et al.* [8], but different from those of Rico M.C. *et al.* [22, 23], possibly due to the tissue-different contexts. Besides, TSP-1 may activate TGF-β1 and its downstream CTGF by binding to latency-associated peptide (LAP) and alter the conformation of TGF-β1[6]. Interestingly, Greenaway *et al.* showed that TSP-1 had a context-dependent direct inhibitory effect on VEGF [23]. Therefore the impact of TSP-1 on activating TGF-β1, CTGF and VEGF in the RA synovium needs to be thoroughly explored further. In summary, our results together with findings from other literatures suggest that there may be a multiple feedback loop among those four proteins (Supplementary Figure 7). In this functional module (i.e. a focused gene regulatory network), the expression of a single angiogenic factor tightly co-related with other factors. Besides, this was also confirmed *in vivo* in the synovial tissue of joints from CIA mice. These angiogenic factors may coordinate to play an important role in the angiogenesis of RA. Therefore, precise-treatments of targeting these proteins could lead to RA disease amelioration [24, 25].

In this study, we also found that TSP-1, TGF-β1, CTGF and VEGF expression levels in either NH-FLS and HDMECs co-culture or RA-FLS and HDMECs co-culture increased significantly after TNF-α induction, in consistent with the report that numerous cytokines, such as IL-6 and MMP-1 from both NH-FLS and RA-FLS, were up-regulated by TNF-α treatment [26, 27]. Further investigations showed that As_2_O_3_ could significantly suppress TNF-α-induced increase of those four proteins in both aforementioned co-cultures, as well as those proteins in RA-FLS and HDMECs co-culture without TNF-α addition, therefore providing a new insight into the anti-angiogenesis activities of As_2_O_3_. Without TNF-α induction, however, little effects were observed in NH-FLS and HDMECs co-cultures under As_2_O_3_ administration. This is similar to effects of As_2_O_3_ in the oncology [28, 29], suggesting As_2_O_3_ may specifically target pathological cells rather than normal cells at such low levels, although such mechanisms remain to be investigated. This favorable anti-angiogenesis profile of As_2_O_3_ is important because it enables formulating drug applications that could exert therapeutic effect without evoking significant deleterious effects on normal cells.

Moreover, further understanding could benefit RA precision therapy. Anti-TNF biologics have proven to be effective in the treatment of RA; however, we speculated that the heterogeneity of RA patients causes many patients’ poor responses to the biologic treatments [30], additionally, the treatment is barely accessible to some patients [31], or they are excluded by a pre-existing malignancy. As_2_O_3_ is attractive to the whole world as one of the most promising broadly effective medications against cancer. It also appears to be a novel promising therapeutic agent for autoimmune disease [32]. The great significance of using the clinical potentials of As_2_O_3_ is that it can “kill two birds with one stone” by inhibiting TNF-α to improve RA and possible RA-cancer comorbidity. However, this current study demonstrated that As_2_O_3_ administration can relieve the severity of murine CIA alongside targeting angiogenesis. After TNF-α was added to a RA-FLS and HDMECs co-culture, TNF-α promoted the expression of angiogenic factors and, subsequently, vascularity. Remarkably, As_2_O_3_ effectively induced an anti-angiogenesis function by suppressing the expression of TSP1, TGF-β1, CTGF and VEGF in a dose-dependent manner. The action of As_2_O_3_ was also shown through significantly improved arthritis in the CIA model in a dose-dependent manner via rescue of the deregulated expression of tissue angiogenic factors. MVD was significantly higher in the synovial tissue of CIA mice than in normal synovial membranes but it was decreased by As_2_O_3_. In summary, As_2_O_3_ can minimize the angiogenesis through inhibiting the TTCV functional module (Supplementary Figure 7). We now continue to research how such factors beyond their quantitative range can lead to failure of the robustness of the module, and ultimately entire system failure as a result of complex diseases such as RA, for which the quantification of factors matter. Besides, As_2_O_3_ binding proteins and novel targets need to be identified in RA [33] to fully understand its effects, and the precise mechanism of As_2_O_3_ treatment on RA angiogenesis warrants further study. Some studies have reported that specific inhibition of NF-κB activity potentiates TNF-α. We need also move forward to determine how this functional module crosstalks with others such as NF-κB-related apoptosis [12] as well as mTOR/PPAR γ-related autophagy involved in RA system failure [34]. Of certain, we are making efforts to lower its potential side effects further via chemical modifications and innovation of drug delivery.

In closing, we observed that the TTCV functional module was systematically up-regulated in the RA synovial tissues both *in vitro* and *in vivo*. Moreover, As_2_O_3_ may have an anti-angiogenesis effect on RA-FLS and the synovium in CIA mice through this functional module, and may serve as a valuable agent for the development of novel RA precision therapeutic strategies, either as monotherapy or in combination therapy.

## MATERIALS AND METHODS

### Cell culture

RA-FLS and NH-FLS were purchased from Cell Applications (San Diego, CA, USA) and were maintained in synoviocyte growth medium (Cell Applications). HDMECs were purchased from ScienCell Research Laboratories (6076 Corte Del Cedro, Carlsbad, CA, USA) and were maintained in Endothelial cell Growth Medium (EGM)-2 from the same company. All cells were maintained in a humidified atmosphere of 5% CO_2_ at 37^°^C and cells of passages from three to five were used for the experiment.

### Co-culture of FLS and HDMECs

Co-culture of FLS and HDMECs was performed in six-well culture plates. RA-FLS and NH-FLS (4×10^5^ cells/ml) were seeded in DMEM supplemented with 10% FBS respectively and allowed to adhere overnight. Cells were then washed with serum-free DMEM and suspensions of HDMECs (ranging from 2×10^5^ cells/ml to 3×10^5^ cells/ml) were added into the upper chamber of a transwell apparatus (Costar, New York, USA). We also used inflammatory cytokines to stimulate the NH-FLS and RA-FLS following protocols described earlier [8]. In brief, stimulation was performed as follows: NH-FLS and RA-FLS were co-cultured with HDMECs respectively, and then each FLS and HDMECs co-cultures were maintained in the presence or absence of TNF-α (100 ng/ml, Peprotech, Rocky Hill, USA) in 1% FBS DMEM. At the same time, various concentrations of As_2_O_3_(0, 0.5 μM, 1.0 μM and 2.0 μM; Yitaida Pharmaceutical Factory, Harbin, Heilongjiang, China) were administered to the co-cultures. After culture in 1% FBS DMEM for 48 h, the supernatants were harvested and centrifuged with 1000 rpm to remove cellular debris. Part of the cell-free culture supernatants were taken out while fresh for the subsequent transwell assays, tube formation assays and *ex vivo* aortic ring angiogenesis assay. Remaining supernatants were frozen at -20^o^C until analysis by commercial ELISA kits for CTGF (KT-50296, Kamiya Biomedical Company, Seattle, WA, USA), TGF-β1 (DB100B, R&D Systems, Minneapolis, USA), TSP-1 (DTSP-10, R&D Systems, Minneapolis, USA) and VEGF (DVE00, R&D Systems, Minneapolis, USA) according to the manufacturers’ protocols. All reactions were conducted in triplicate.

### Transwell assays

The lower chamber was a 24-well culture plate (BD Falcon, New Jersey, USA) containing 600 µL of the supernatants from the co-cultures. Inserts (upper chambers) membranes with 8.0 μm pore size (Costar, New York, USA) were then placed in the lower chambers as described before [19]. HDMECs (3×10^4^ cells in 200 µL serum-free DMEM) were added to each upper chamber and incubated for 6 h. Non-migrating cells on the upper surface of the filter were removed with a cotton swab. Cells that migrated to the lower phase of the upper chamber were then fixed in methanol for 30 minutes and stained with crystal violet for 30 minutes at room temperature. Images were obtained via microscopy, and the cell number was quantified by counting the number of cells that migrated to the lower side of the filter using optical microscopes (LEICA DMi8) by 100× magnification. Six optical fields were counted for each assay. Each assay was conducted with three wells and similar experiments were repeated at least three times.

### HDMECs tube formation *in vitro*

In this assay, 50 μL Matrigel (Corning, New York, USA) was plated in a 96-well culture plate after thawing on ice and allowed to promote gelling for 30 minutes at 37^°^C in humidified air with 5% CO_2_, then 50 μL of fresh supernatant from the co-culture was added to each well. HDMECs were then trypsinized and re-suspended at a concentration of 2×10^5^ cells/ml in DMEM. Next, 100 μL of cell suspension was added to each chamber. After 6 h of incubation, endothelial cell tube formation was assessed using phase-contrast microscopy and then photographed. The morphology of the tube-like structures in the well were assessed and quantified by a blinded observer, who counted the number of intersections between branches of the endothelial cell networks in the entire field. The tube analysis was determined from six randomly chosen fields (50× magnification). The assay was performed in triplicate.

### Mouse aortic ring angiogenesis assay

The *ex vivo* aortic ring assay was performed as previously described [35]. Briefly, 24-well culture plates were coated with 200 μL growth factor-reduced (GFR) Matrigel (Corning, New York, USA). Thoracic aortas were removed from 6- to 8-week-old C57BL/6 mice (SPF, Laboratory Animal Center of the Second Affiliated Hospital of Harbin Medical University) and sectioned into 1-mm-long aortic rings. Each ring was placed in the pre-coated well and covered with an additional 200 μl of Matrigel. Aortic rings were incubated for 3 days in 1% FBS EGM-2. After verifying sprouts from the aortic rings, the EGM-2 was replaced with 500 μL of fresh supernatants from NH-FLS and HDMECs co-culture as well as RA-FLS and HDMECs co-culture treated or untreated with TNF-α or As_2_O_3_ and the aortic rings were further incubated for 3 days. Microvessel outgrowths were examined with microscope (LEICA DMi8) and photographed (50× magnification). The area of outgrowth was analyzed using Image J software. All assays were performed by using 3 aortic rings per sample.

### Stimulation

RA-FLS were seeded onto six-well plates (5×10^5^ cells/well) for 24 h and treated with a fresh culture medium containing recombinant human CTGF (Prospec-Tany TechnoGene Ltd, Ness-Ziona, Israel), TSP-1 (Prospec-Tany TechnoGene Ltd, Ness-Ziona, Israel), TGF-β1 (sigma, St.Louis, MO, USA) and VEGF (Peprotech, Rocky Hill, USA) at concentrations of 500 ng/ml [2], 1000 ng/ml [8], 5 ng/ml [8] and 50 ng/ml [36] respectively. RNA of FLS and supernatants containing proteins were harvested from the cells 48 h after addition for evaluation of TSP-1, TGF-β1, CTGF and VEGF stimulation.

### RNA interference analysis

TSP-1, TGF-β1, VEGF and CTGF knockdown experiments were performed by transfecting specific TSP-1, TGF-β1, VEGF and CTGF small interfering RNA (siRNA) into RA-FLS. The siRNA sequences targeting TSP-1 were: forward, 5′-GGAGUUCAGUACAGAAAUATT-3′ and reverse, 5′-UAUUUCUGUACUGAACUCCTT-3′. The siRNA sequences targeting TGF-β1 were: forward, 5′-CCCACAACGAAAUCUAUGATT-3′ and reverse, 5′-UCAUAGAUUUCGUUGUGGGTT-3′. The siRNA sequences targeting CTGF were: forward, 5′-CCCGGGUUACCAAUGACAATT-3′ and reverse, 5′-UUGUCAUUGGUAACCCGGGTT-3′. The siRNA sequences targeting VEGF were: forward, 5′-UGGAGUGUGUGCCCACUGATT-3′ and reverse, 5′-UCAGUGGGCACACACUCCATT-3′. FLS were plated in six-well plates at a density of 5×10^5^ cells per well with antibiotic-free DMEM supplemented with 10% FBS and grown to 50-70% confluence and transfected with siRNA using Lipofectamine 2000 (Invitrogen, Carlsbad, CA, USA) according to the manufacturer’s protocol. The final concentration of siRNA duplex was 80 nM. At 48 h after transfection, 80% knockdown efficiency was achieved, as determined by real- time PCR and western blot analysis. RNA of FLS and supernatants containing the proteins were harvested from the cells 48 h after transfection for further experiments. Transfection was performed in multiple individual wells (*n* = 3). The experiments were repeated at least three times.

### RNA preparation and real-time quantitative PCR analysis

Total RNA was extracted from FLS using Trizol reagent (Invitrogen, Carlsbad, CA, USA) and dissolved in DEPC-treated distilled water. The concentration of total RNA was determined with NanoVue Plus. RNA were subsequently converted to cDNA using the Prime Script RT reagent kit (TaKaRa Biotechnology, Dalian, China) according to the manufacturer’s protocol and subjected to real-time quantitative PCR amplification using the GoTaq qPCR Master Mix (Promega, Beijing, China). Specific amplification was performed using the primers of *TSP-1* genes (forward primer: 5′-GCCTGATGACAAGTTCCAAGAC- 3′; reverse primer: 5′-GACACCACGCTGAAGACCTG-3′), *TGF-β1* genes (forward primer: 5′-CTACTACGCCAAGGAGGTCAC-3′; reverse primer: 5′- GAGGTATCGCCAGGAATTGTTG-3′), *CTGF* genes (forward primer: 5′-CTTGCGAAGCTGACCTGGAA- 3′; reverse primer: 5′-AGCTCAAACTTGATAGGCTTGGAG -3′) and *VEGF* genes (forward primer: 5′-TTCTGGGCTGTTCTCGCTTC- 3′; reverse primer: 5′- CTCTCCTCTTCCTTCTCTTCTTCC-3′). GAPDH (forward primer: 5′-ATGGTGGGTATGGGTCAGA AG-3′; reverse primer: 5′-TGGCTGGGGTGTTGAAGGTC-3′) was used as an internal control. All samples were run in triplicate and the results were evaluated using the 2^-ΔΔ CT^ method as previously described [34].

### Western blot analysis

The knockdown efficiency of TSP-1, TGF-β1, CTGF and VEGF was determined by western blot analysis, including anti-Thrombospondin antibody (catalog no.ab85762, Abcam, Cambridge, MA), anti-TGF-β1 antibody (catalog no.ab9758, Abcam, Cambridge, MA), anti-CTGF antibody (catalog no.ab6992, Abcam, Cambridge, MA) and anti-VEGF antibody (catalog no.ab115961, Abcam, Cambridge, MA). Equal amounts of protein extract (50μg per lane) were separated by sodium dodecyl sulfate polyacrylamide gel electrophoresis as described before [34]. The antibodies against specific proteins were then added.

### Animal model and experimental protocol

Male DBA/1J mice (8-week-old and 20g ± 2g in weight, SPF, SLAC, Hunan, China) were housed with free access to feed and water. All animal experiments were performed according to the National Institutes of Health Guide for the Care and Use of Laboratory Animals, and the protocols were approved by the Institutional Animal Care and Use Committee of Harbin Medical University. CIA mouse model (*n* = 6 per experimental group) was generated by referring to reported method. Briefly, bovine type II collagen (CII, Chondex, Redmond, WA, USA) was emulsified in an equal volume of complete Freund’s adjuvant (CFA) containing 4 mg/ml heat-killed H37RA (Chondex, Redmond, WA, USA). Each DBA/1J mouse was given a 100μl of the emulsion containing 100μg CII via intradermally injection at the base of the tail. On Day 21 after primary immunization, the mice received a booster shot with 100μg CII emulsified with an equal volume of incomplete Freund’s adjuvant (Chondex, Redmond, WA, USA). After induction of arthritis (on Day 21), the mice were divided into the following groups (six mice in each group): CIA control (CIA without treatment), As_2_O_3_ (CIA treated with As_2_O_3_ at doses of 1.0, 2.0 and 5.0 mg/kg/day), MTX (CIA treated with MTX at a dose of 1.5 mg/kg/week, as a treatment control) and a normal control group (without immunization). For evaluation of As_2_O_3_ toxicity, As_2_O_3_ (normal mice treated with As_2_O_3_ at doses of 1.0, 2.0 and 5.0 mg/kg/day) groups were also performed with six mice in each group. Mice under treatment were administrated As_2_O_3_ and MTX intraperitoneally from Day 26 to Day 39 after the first immunization. Normal and CIA model mice were given an equal volume of PBS. The mice were carefully monitored for symptoms of toxicity, and weighed every three days. The mean body-weight of each group was calculated and used as a parameter of toxicity, as described previously [37].

### Assessment of arthritis severity

Clinical arthritis was assessed daily from Day 19 after primary immunization and arthritic score was evaluated every 3 days to the end of experiment period. To quantitatively evaluate the severity of the arthritis, a scoring system was used according to a modified version of a standard protocol [38]. Briefly, the severity of arthritis of the four paws were assessed by using the following scale from 0 to 4: 0 = no evidence of erythema and swelling; 1 = erythema and mild swelling confined to the tarsals or ankle joint; 2 = erythema and mild swelling extending from the ankle to the tarsals; 3 = erythema and moderate swelling extending from ankle to metatarsal joints; 4 = erythema and severe swelling encompass the ankle, foot and digits, or ankylosis of the limb. Each limb was graded, giving a maximum score of 16 per animal. Mice with clinical score greater than 4 were given a diagnosis of arthritis.

### Histological analysis

After sacrifice, whole knee joints of mice were collected and fixed in 10% buffered neutral formalin and decalcified in 15% EDTA (pH 7.4) for up to 5 weeks. The knees were embedded in paraffin, cut into 5 μm sections, and stained with H&E. To quantitatively evaluate the severity of the arthritis, a scoring system was employed referring to the reported protocol [39, 40]. In brief, histological changes were examined by microscopic evaluation and scored in a blinded manner by two independent observers based on cell infiltration, cartilage destruction and bone erosion parameters as follows: 0 = normal joint structure; 1 = mild changes, synovitis, and pannus formation with few discrete cartilage focal erosions; 2 = moderate changes, accompanying loss of large areas of cartilage, eroding pannus formation, and synovial hyperplasia with infiltrating inflammatory cells; 3 = severe synovitis, cartilage and bone erosion, and destruction of joint architecture.

### Immunohistochemical analysis

The immunohistochemical analysis was performed as previously described with some modifications[34]. Knee joint sections on slides were incubated with anti-TSP-1, TGF-β1, CTGF, VEGF and von Willebrand factor (vWF) antibody (BA2130, BA0290, BA0752, BA0407, BA0046, Boster, Wuhan, China). Subsequently, the sections were stained using polymer HRP detection system (PV9001, ZSGB-BIO, Beijing, China) and were visualized with DAB peroxidase substrate kit (ZLI-9017, ZSGB-BIO, Beijing, China). Rabbit anti-mouse vWF was used to identify endothelium as described before [41]. Following immunostaining, the synovium area in the joint of each section was evaluated under a microscope (LEICA DMi8) in three randomly selected areas at a magnification of 400×. The number of positively stained cells and total cells were counted by two different observers, and the means of the ratio of these two groups of cells were calculated. The number of microvessels positively stained with anti-vWF was also counted in the same area on the next serial section.

### Statistical analysis

The data were expressed as means ± S.E.M. Statistical analysis was conducted with paired t test or ANOVA as appropriate by using SPSS version 17.0 software*.* Values of *p* < 0.05 were considered statistically significant.

### Ethical approval

Harbin Medical University Ethics Committee Board.

## SUPPLEMENTARY MATERIALS FIGURES


