# Supplementary material for: Inhibition of angiogenesis by arsenic trioxide via TSP-1–TGF-β1-CTGF–VEGF functional module in rheumatoid arthritis
Source: Oncotarget. 2017 Aug 3;8(43):73529–46. doi: 10.18632/oncotarget.19867 (PMC5650279; doi:10.18632/oncotarget.19867)
Supplement: Supplementary file 1 [file oncotarget-08-73529-s001.pdf]

Inhibition of angiogenesis by arsenic trioxide *via* TSP-1–TGF-β1-CTGF–VEGF functional module in rheumatoid arthritis

SUPPLEMENTARY MATERIALS

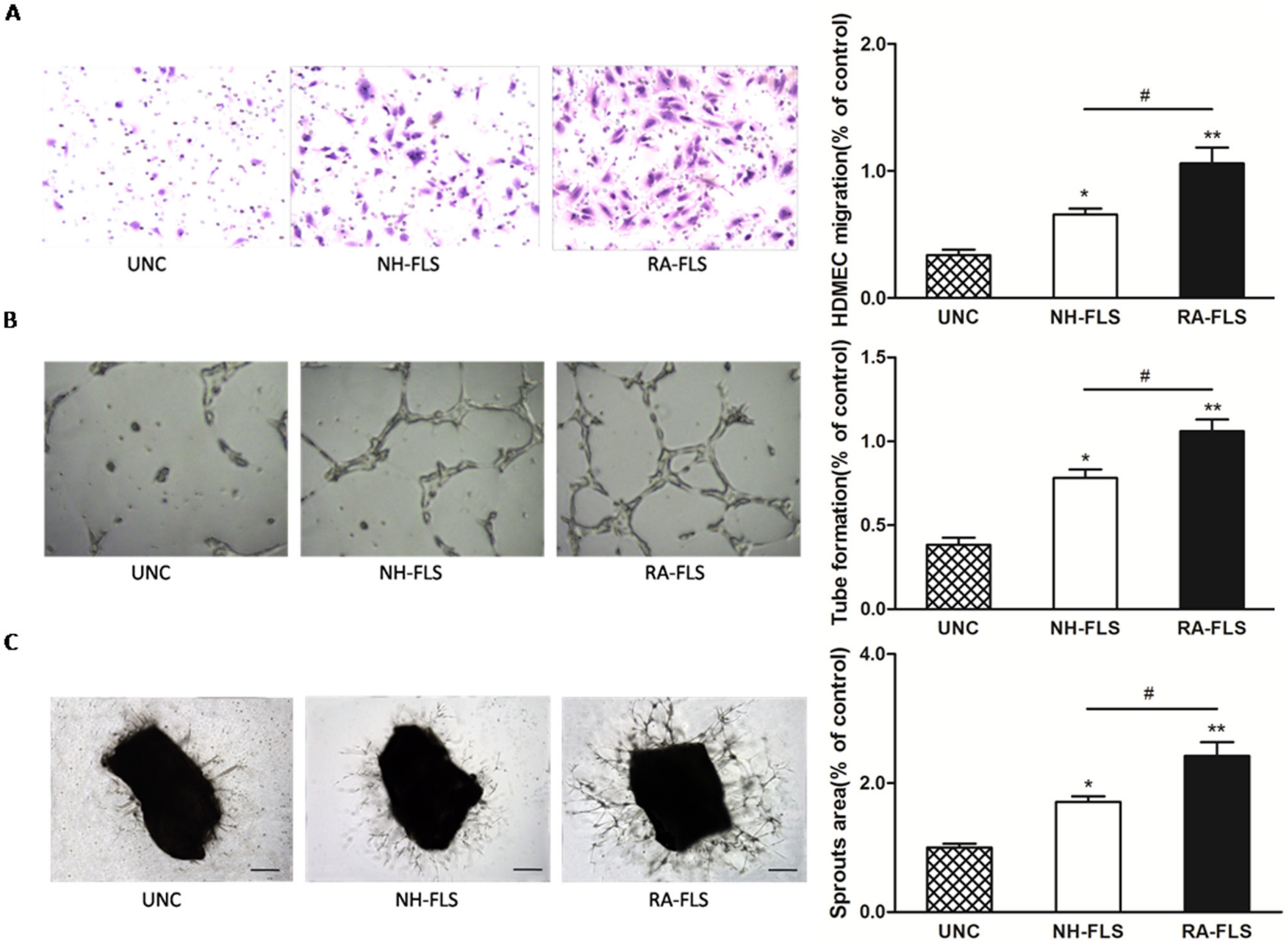

Supplementary Figure 1: Results of transwell assay, tubule formation test and *ex vivo* aortic ring angiogenesis assay along with unconditioned media alone as well as supernatants of each FLS and HDMECs co-cultures. Normal human (NH) FLS and rheumatoid arthritis (RA) FLS were co-cultured with HDMECs for 48 h, respectively. **(A and B)** Transwell assay (A; n=3) and tube formation test (B; n=3) for 6 h demonstrated significant up-regulation in migration and capillary-like structure formation of HDMECs respectively under treatment of supernatants from each FLS and HDMECs co-cultures (n=3, respectively) compared to those under treatment of unconditioned media (n=3, respectively; *p<0.05, **p<0.01), and migration and tube formation of HDMECs increased significantly with treatment of supernatants from RA-FLS and HD-MECs co-culture (n=3) compared to those from NH-FLS and HDMECs co-culture (n=3; #p<0.05). **(C)** Mouse aortic rings were placed on GFR-Matrigel-coated plates and incubated in 1% FBS EGM-2. On 3rd day, the EGM-2 were exchanged with supernatants from FLS and HDMECs co-cultures and further incubated for 3 days. *Ex vivo* aortic ring angiogenesis assay showed significant up-regulation in microvessel sprouting under treatment of supernatants from each FLS and HDMECs co-cultures (n=3, respectively) compared to those in unconditioned media (n=3; *p<0.05, **p<0.01), and microvessel out-growth increased significantly under application of supernatants from RA-FLS and HDMECs co-culture (n=3) compared to those from NH-FLS and HDMECs co-culture (n=3; #p<0.05). Bars = 300μm. Original magnification = ×5. Results are expressed as the mean ± S.E.M. UNC = unconditioned medium control. As shown in Fig.1, the migration, tube formation of HDMECs and microvessel sprouting increased significantly with treatment of supernatants from RA-FLS and HDMECs co-culture compared to those from NH-FLS and HDMECs co-culture.

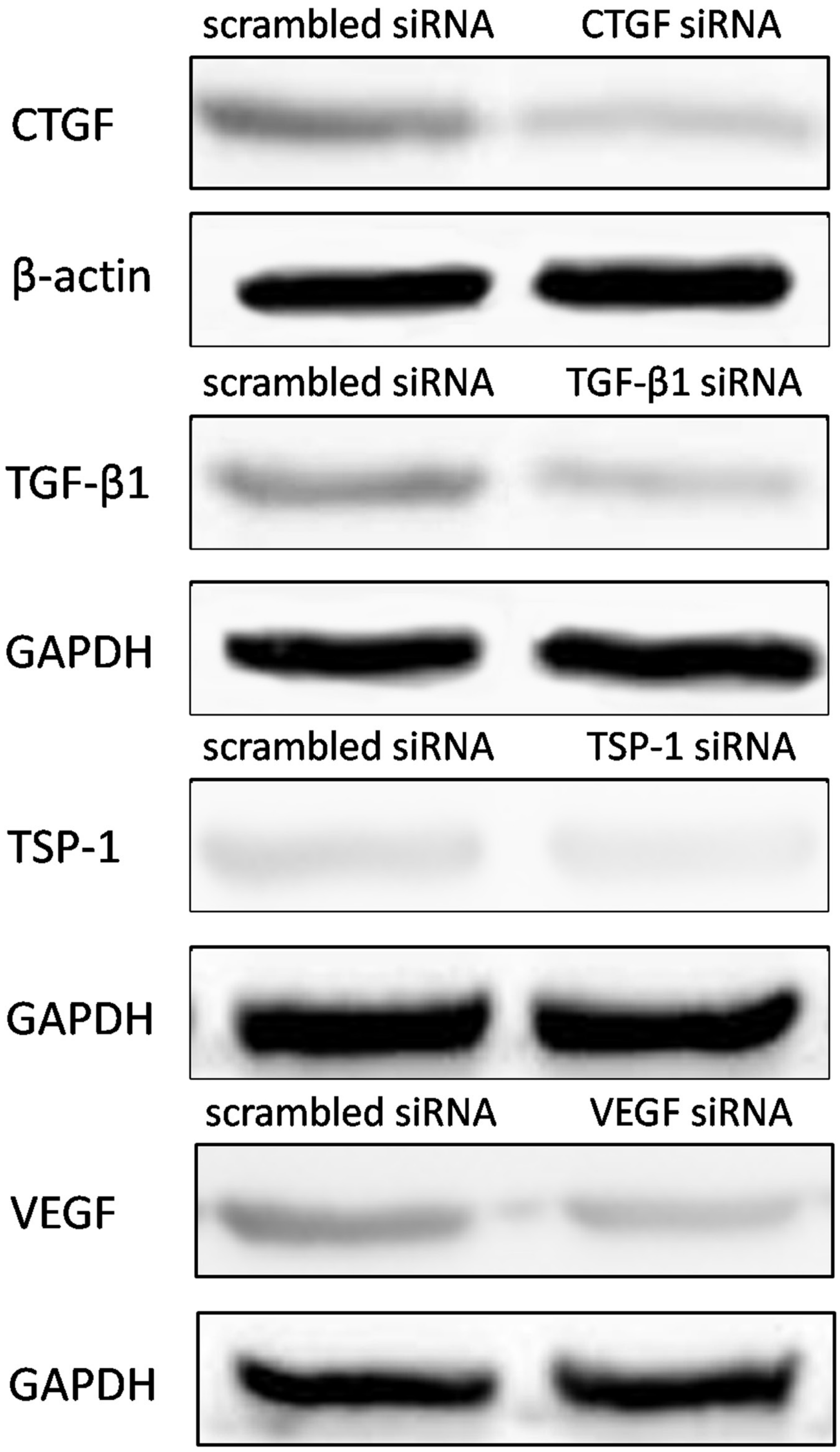

Supplementary Figure 2: Silencing efficiency of TSP-1, TGF-β1, CTGF and VEGF siRNAs in RA-FLS (n=3) was confirmed by real-time PCR and western blotting analysis. Significant decrease in the expression of TSP-1, TGF-β1, CTGF and VEGF were observed at both mRNA (Fig.2B) and protein levels (this picture) in TSP-1, TGF-β1, CTGF and VEGF siRNA-treated cells compared to scrambled siRNA-treated cells. Three sets of siRNAs for each gene have been initially checked and the results from most efficient ones are presented.

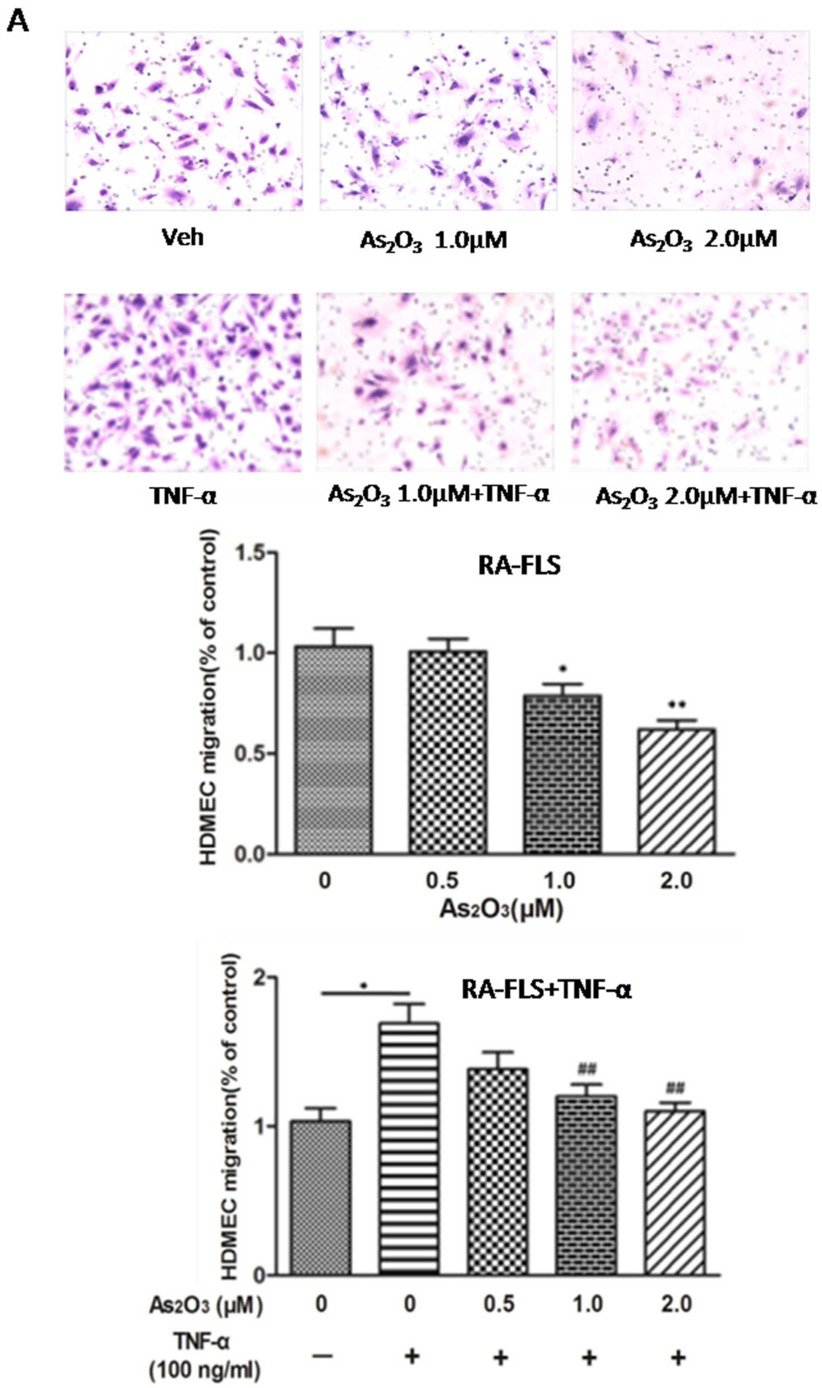
A
Veh
As2O3 1.0μM
As2O3 2.0μM
TNF-α
As2O3 1.0μM+TNF-α
As2O3 2.0μM+TNF-α
RA-FLS
HDMEC migration(% of control)
As2O3(μM)
RA-FLS+TNF-α
As2O3 (μM)
TNF-α
(100 ng/ml)

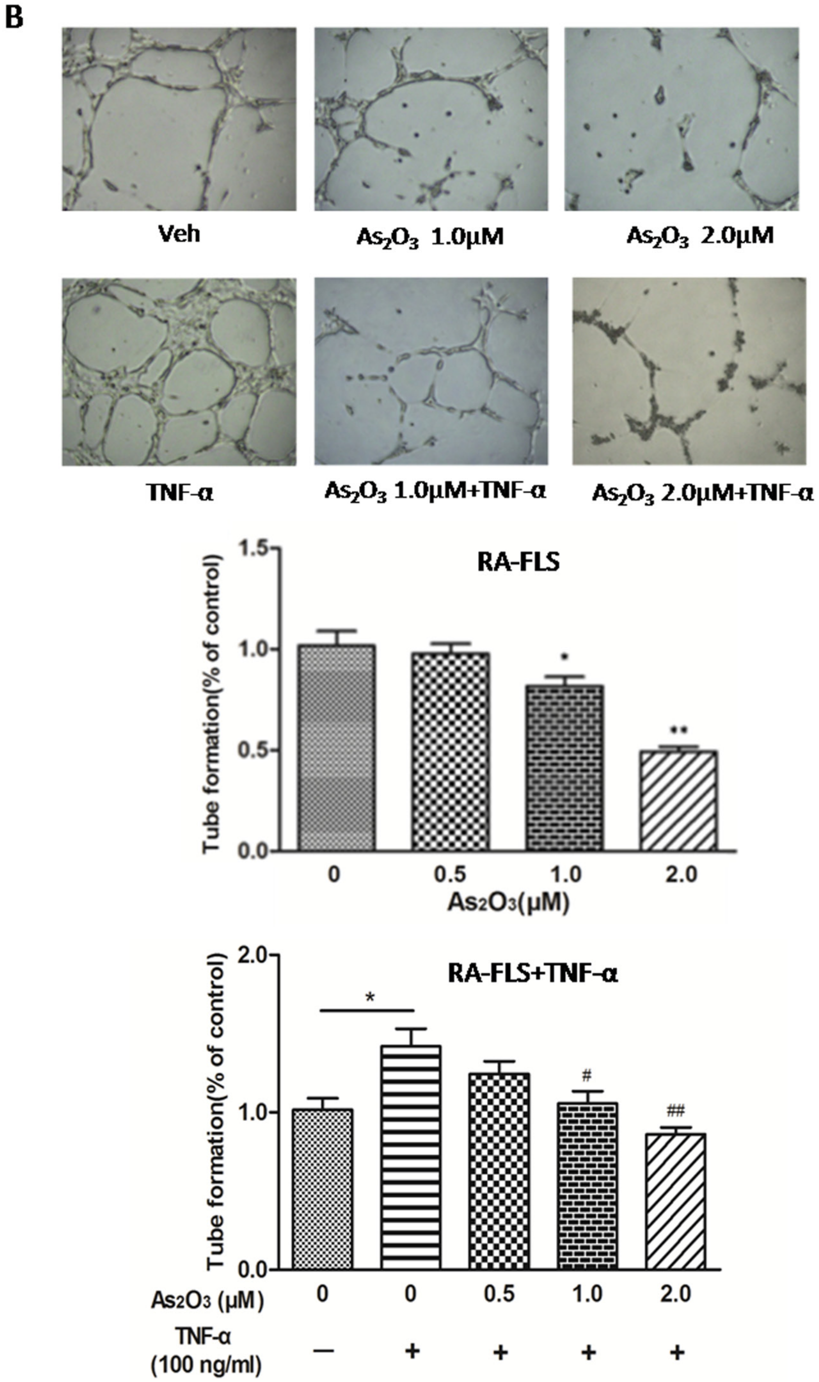
B
Veh
As2O3 1.0μM
As2O3 2.0μM
TNF-α
As2O3 1.0μM+TNF-α
As2O3 2.0μM+TNF-α
RA-FLS
Tube formation(% of control)
1.5
1.0
0.5
0.0
0
0.5
1.0
2.0
As2O3(μM)
RA-FLS+TNF-α
Tube formation(% of control)
2.0
1.0
0.0
As2O3 (μM)
TNF-α
(100 ng/ml)

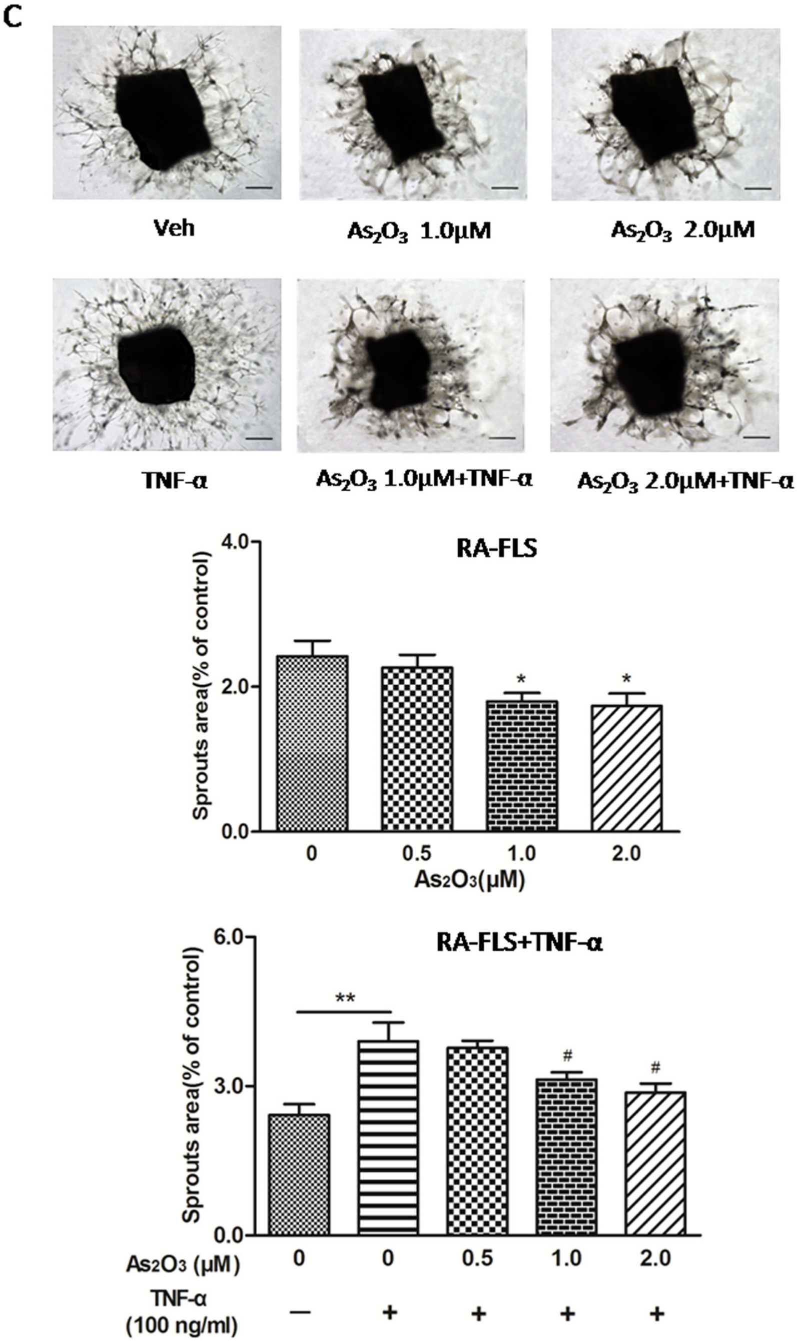

Supplementary Figure 3: As_2O_3 attenuates the angiogenesis *in vitro* and *ex vivo* systems under treatment of media from RA-FLS and HDMECs co-cultures. Hereby we describe the effect of As_2O_3 at a dose of 1.0 μM, and other data were also demonstrated in Fig.3 in main text. RA-FLS and HDMECs co-cultures were respectively treated with As_2O_3 alone or together with TNF-α (100ng/ml) for 48 h. **(A and B)** Transwell assay and tube formation test were performed by applying supernatants from RA-FLS and HDMECs co-cultures to HDMECs for 6 h respectively. HDMECs migration and tube formation were increased by supernatants from RA-FLS and HDMECs co-culture under TNF-α treatment (n=3; **p*<0.05), while As_2O_3 at doses of 1.0 μM (n=3) and 2.0 μM (n=3) played a significantly opposite role in migration and tube formation with or without TNF-α (**p*<0.05, ***p*<0.01, #*p*<0.05, ##*p*<0.01), which were also in a dose dependent manner. **(C)** *Ex vivo* aortic ring angiogenesis assay showed similar changes of microvessel sprouting as demonstrated in migration and tube formation of HDMECs in the transwell assay and tube formation test above (n=3; **p*<0.05, ***p*<0.01, #*p*<0.05). Bars=300μm. Original magnification = ×5. Results are expressed as the mean ± S.E.M. **p*<0.05, ***p*<0.01 versus Veh, #*p*<0.05, ##*p*<0.01 versus TNF-α. Veh = vehicle control under treatment of 1% FBS DMEM alone. TNF-α = control group under treatment of TNF-α (100ng/ml) only.

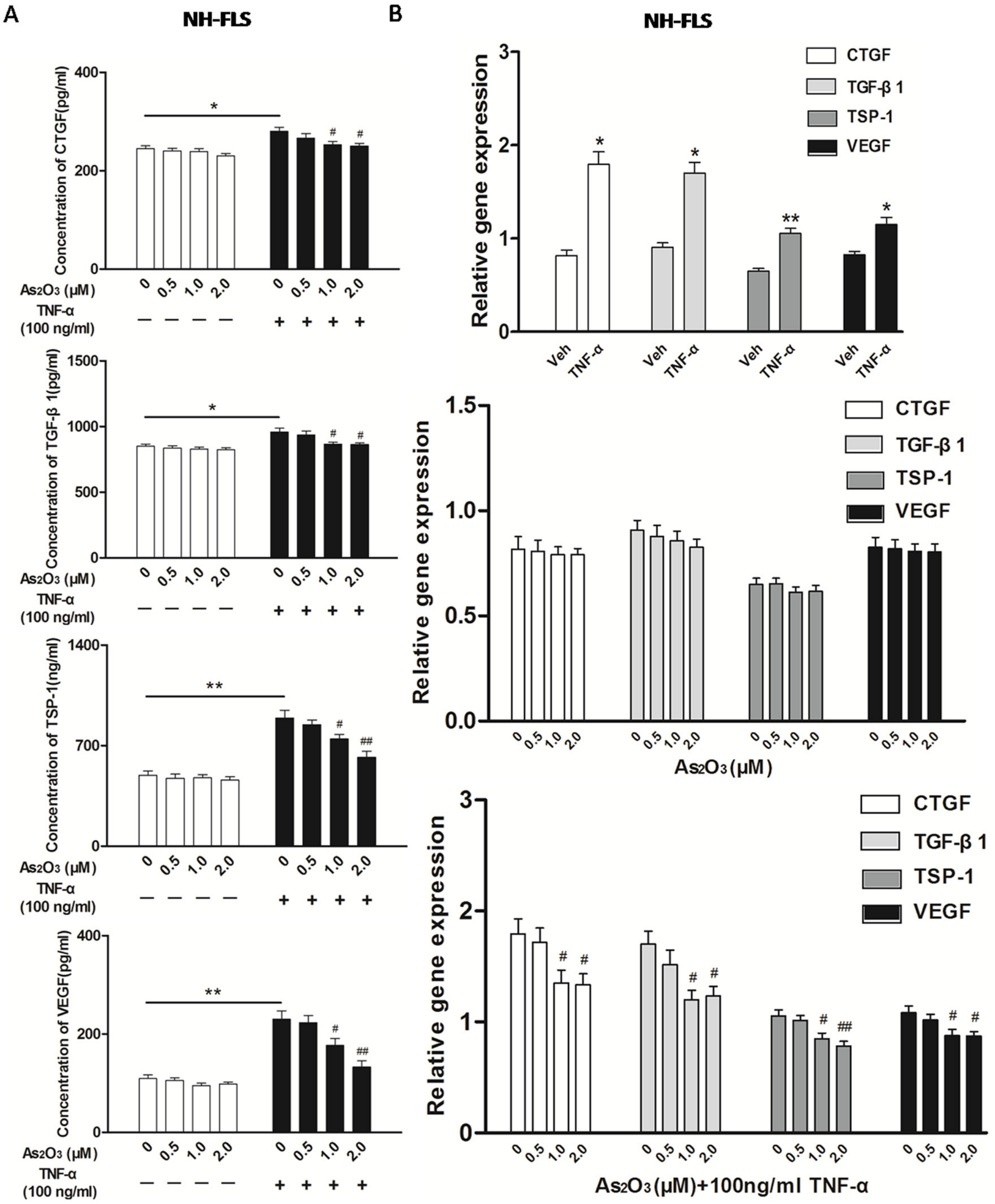

Supplementary Figure 4: The effect of As_2O_3 treatment on TSP-1, TGF-β1, CTGF and VEGF expression in NH-FLS. NH-FLS and HDMECs co-cultures were treated with As_2O_3 alone or together with TNF-α (100ng/ml) for 48 h. (A) TSP-1, TGF-β1, CTGF and VEGF protein expression in the supernatants from NH-FLS and HDMECs co-cultures were analyzed by ELISA. Results showed that concentrations of TSP-1, TGF-β1, CTGF and VEGF increased significantly after treatment with TNF-α (100 ng/ml) (n=3) compared to vehicle control group (n=3; *$p<0.05$, **$p<0.01$), and then significantly decreased concentrations of TSP-1, TGF-β1, CTGF and VEGF were observed after the treatment of As_2O_3 at doses of 1.0 µM and 2.0 µM in a dose dependent manner (n=3, respectively; #$p<0.05$, ##$p<0.01$). However no significant decreased expression of those proteins were seen in supernatants from NH-FLS and HDMECs co-cultures under As_2O_3 treatment only (n=3, respectively). (B) TSP1, TGF-β1, CTGF and VEGF mRNA expression in co-cultured NH-FLS were performed by real-time PCR. Results showed similar changes of TSP-1, TGF-β1, CTGF and VEGF mRNA expression as demonstrated in protein regulation after treatment of As_2O_3 alone (n=3) or together with TNF-α (100ng/ml) (n=3; *$p<0.05$, **$p<0.01$, #p<0.05, ##$p<0.01$). Results are expressed as the mean ± S.E.M. *$p<0.05$, **$p<0.01$ versus Veh, #$p<0.05$, ##$p<0.01$ versus TNF-α. Veh = vehicle control under treatment of 1% FBS DMEM alone. TNF-α = control group under treatment of TNF-α (100ng/ml) only.

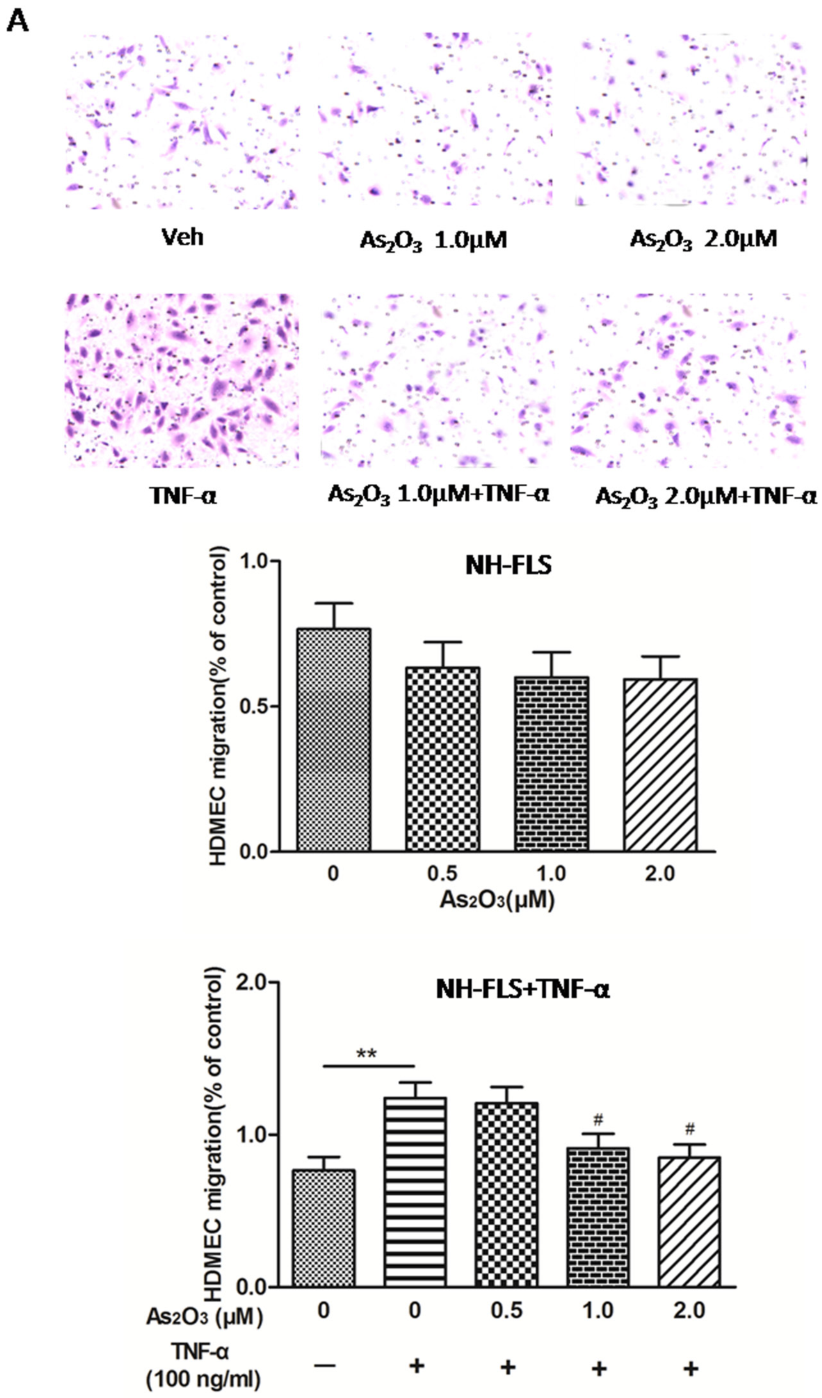

A
Veh
As2O3 1.0μM
As2O3 2.0μM
TNF-α
As2O3 1.0μM+TNF-α
As2O3 2.0μM+TNF-α
NH-FLS
HDMEC migration(% of control)
1.0
0.5
0.0
0
0.5
1.0
2.0
As2O3(μM)
NH-FLS+TNF-α
HDMEC migration(% of control)
2.0
1.0
0.0
**
#
#
As2O3 (μM)
0
0
0.5
1.0
2.0
TNF-α
(100 ng/ml)
—
+
+
+
+

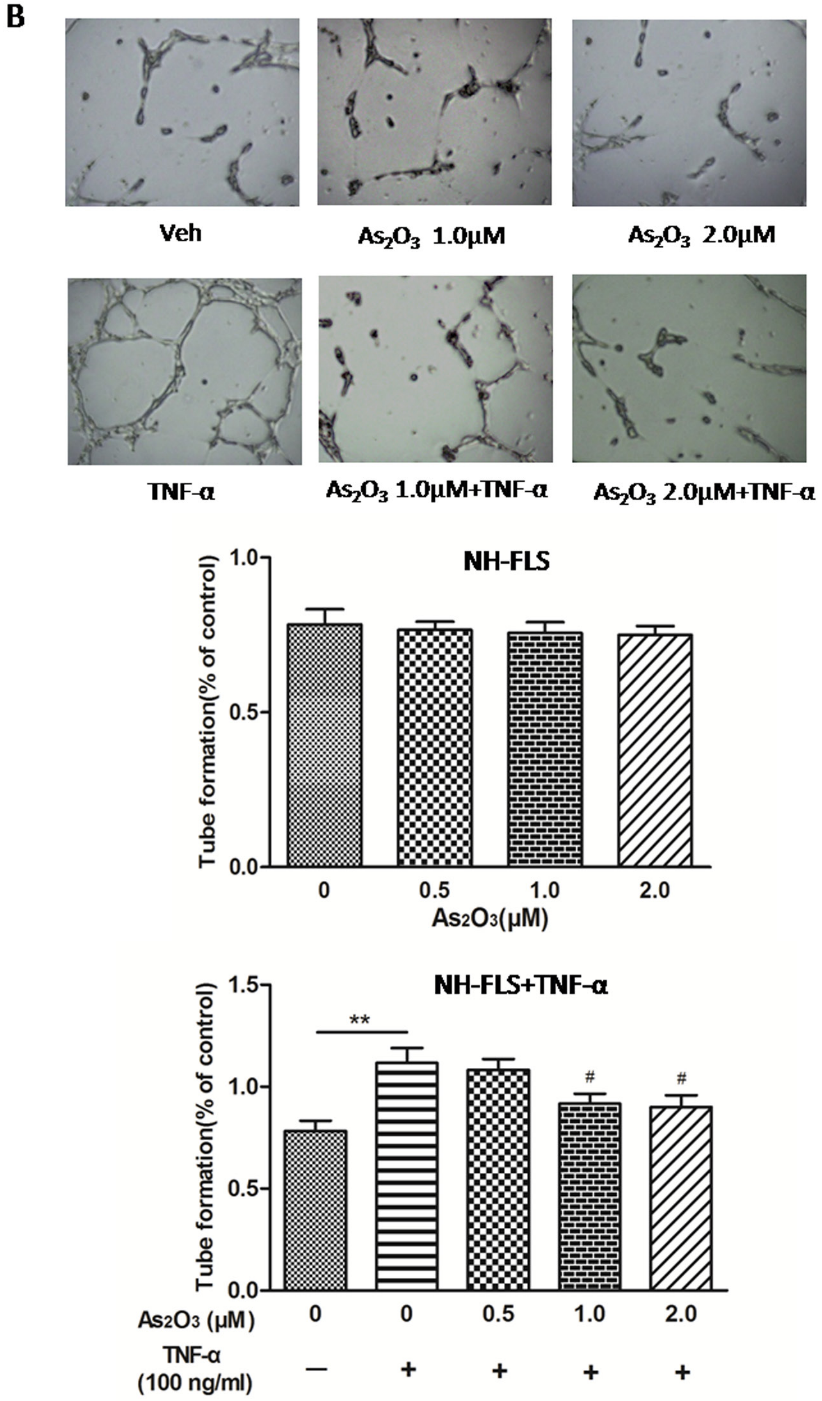
B
Veh
As_2O_3 1.0μM
As_2O_3 2.0μM
TNF-α
As_2O_3 1.0μM+TNF-α
As_2O_3 2.0μM+TNF-α
NH-FLS
Tube formation(% of control)
1.0
0.5
0.0
0
0.5
1.0
2.0
As_2O_3(μM)
NH-FLS+TNF-α
Tube formation(% of control)
1.5
1.0
0.5
0.0
**
#
#
As_2O_3 (μM) 0 0 0.5 1.0 2.0
TNF-α (100 ng/ml) — + + + +

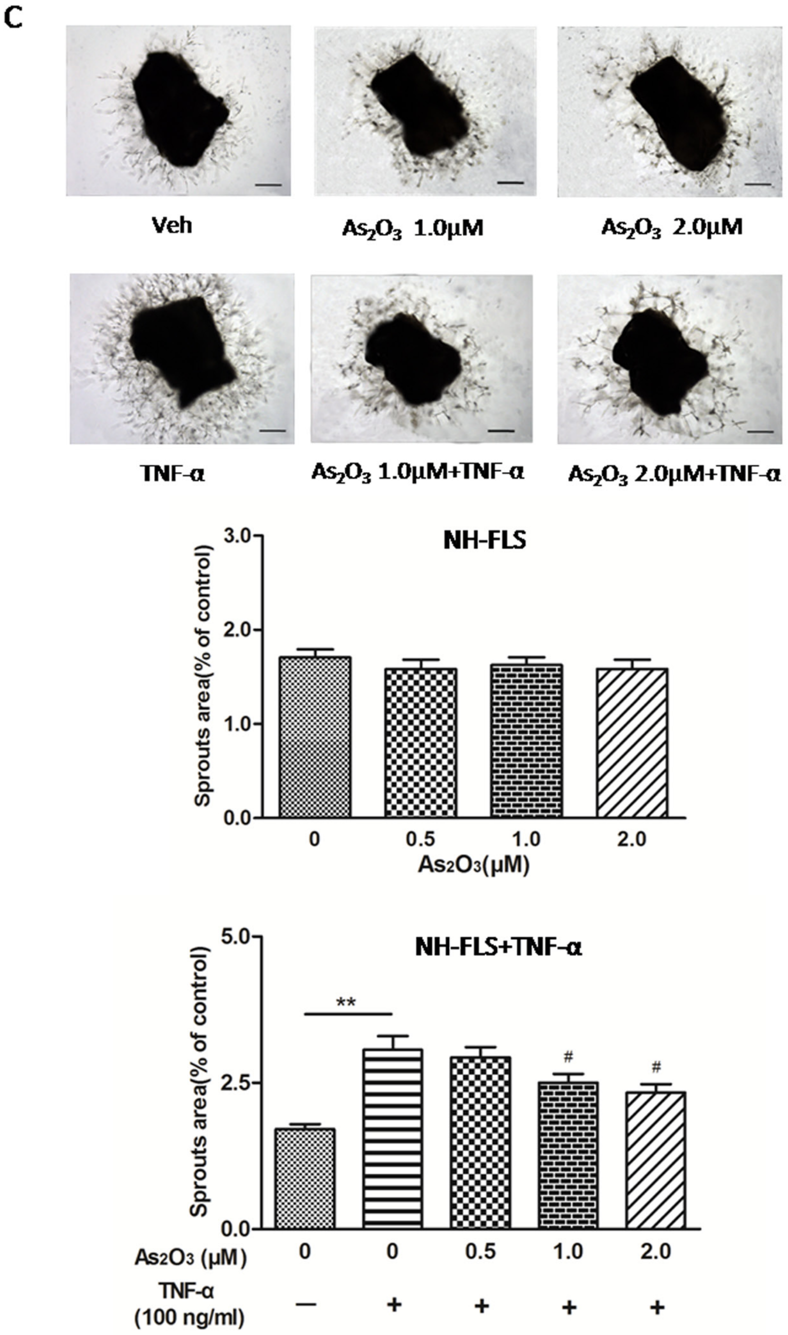

Supplementary Figure 5: The effect of As_2O_3 treatment on angiogenesis *in vitro* and *ex vivo* systems under treatment of media from NH-FLS and HDMECs co-cultures. **(A and B)** Transwell assay and tube formation test were performed by applying supernatants from NH-FLS and HDMECs co-cultures to HDMECs for 6 h respectively. Results showed that migration and capillary-like structure formation of HDMECs significantly increased after TNF-α (100ng/ml) stimulation (n=3, respectively; **p<0.01), while subsequently As_2O_3 at doses of 1.0 μM (n=3) and 2.0 μM (n=3) played a significantly inhibitory role in migration and tube formation (#p<0.05), which were also in a dose dependent manner. However, there was no significant decrease of migration and tube formation in supernatants from NH-FLS and HDMECs co-cultures under As_2O_3 treatment only (n=3, respectively). **(C)** *Ex vivo* aortic ring angiogenesis assay showed similar changes of microvessel sprouting as demonstrated in migration and tube formation of HDMECs above (n=3; **p<0.01, #p<0.05). Bars=300μm. Original magnification = ×5. Results are expressed as the mean ± S.E.M. *p<0.05, **p<0.01 versus Veh, #p<0.05, ##p<0.01 versus TNF-α. Veh = vehicle control under treatment of 1% FBS DMEM alone. TNF-α = control group under treatment of TNF-α (100ng/ml) only.

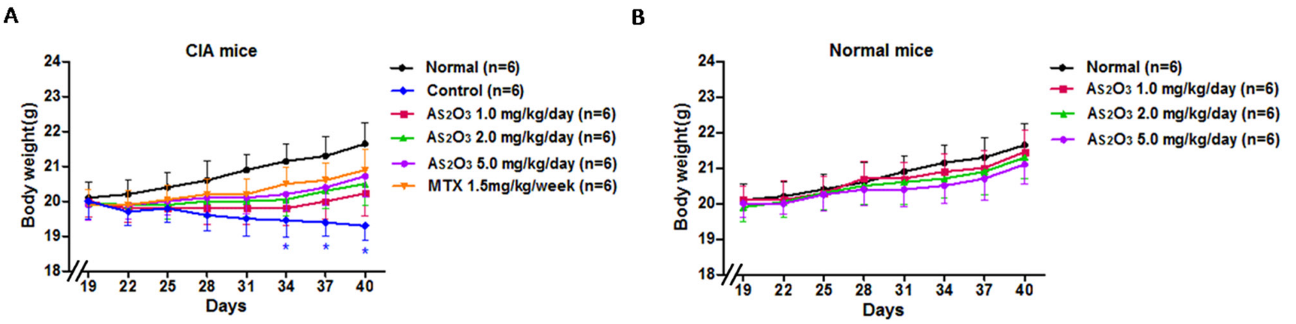

Supplementary Figure 6: As_2O_3 inhibited body-weight loss of CIA mice. (A) Mean body-weight of CIA control group (n=6) decreased significantly compared to normal mice (n=6) from Day 34 to Day 40 (**p*<0.05), however, mean body-weight of groups under As_2O_3 and MTX treatment (n=6, respectively) showed no significant change compared to normal mice (n=6). **(B)** For better evaluation of As_2O_3 toxicity, the assessment for normal mice under different doses of As_2O_3 treatment were performed. No significant body-weight loss was found among different treatments. Results are expressed as the mean ± S.E.M. Normal = normal mice. Control = CIA control mice. MTX = methotrexate.

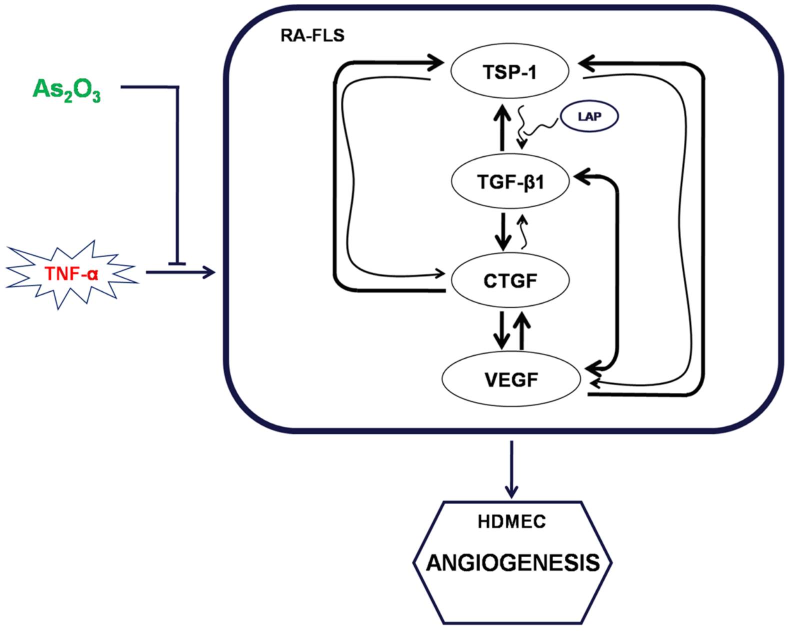

Supplementary Figure7: The regulatory network among TSP-1, TGF-β1, CTGF and VEGF.

The functional module of TSP-1-TGF-β1-CTGF-VEGF (TTCV) is up-regulated in RA-FLS, leading to the angiogenesis in synovium of RA. Our results and other labs' reports [1-10] suggest that there may be a multiple feedback loop among those four functional proteins, and one simplified model is provided here.

TGF-β1 is central in functional module, which leads to the up-regulation of TSP-1, CTGF and VEGF expression. VEGF, which may be induced by TGF-β1 and CTGF, could up-regulate TGF-β1, CTGF and TSP-1 production. Therefore, VEGF is the functional cytokine of the TTCV functional module, leading to the angiogenesis in synovium of RA. Furthermore, CTGF could promote TSP-1 expression. Surprisingly, the expression of TGF-β1, CTGF and VEGF showed no significant change with TSP-1 intervention. TSP-1 may activate latent form of TGF-β1. And TSP-1 had a context-dependent effect on TGF-β1, CTGF and VEGF. TNF-α, which is shown in red, promotes the expression of TSP-1, TGF-β1, CTGF and VEGF in RA-FLS, while As_2O_3, which is shown in green, can effectively suppress the function of TNF-α, representing an anti-angiogenesis effect. Here fold lines indicate our original reports for the first time or results from our study corroborated in other labs; waved lines indicate results having difference between other labs' observation in different tissues (or contexts) and our study.

REFERENCES

1. Wang JG, Xu WD, Zhai WT, Li Y, Hu JW, Hu B, Li M, Zhang L, Guo W, Zhang JP, Wang LH, Jiao BH. Disorders in angiogenesis and redox pathways are main factors contributing to the progression of rheumatoid arthritis: a comparative proteomics study. Arthritis Rheum. 2012; 64:993–1004. https://doi.org/10.1002/art.33425
2. Nakagawa T, Lan HY, Glushakova O, Zhu HJ, Kang DH, Schreiner GF, Böttinger EP, Johnson RJ, Sautin YY. Role of ERK1/2 and p38 mitogen-activated protein kinases in the regulation of thrombospondin-1 by TGF-beta1 in rat proximal tubular cells and mouse fibroblasts. J Am Soc Nephrol. 2005; 16:899–904. https://doi.org/10.1681/ASN.2004080689
3. Tang YN, Ding WQ, Guo XJ, Yuan XW, Wang DM, Song JG. Epigenetic regulation of Smad2 and Smad3 by profilin-2 promotes lung cancer growth and metastasis. Nat Commun. 2015; 6:8230. https://doi.org/10.1038/ncomms9230
4. Rico MC, Rough JJ, Manns JM, Del Carpio-Cano F, Safadi FF, Kunapuli SP, DeLa Cadena RA. Assembly of the prothrombinase complex on the surface of human foreskin fibroblasts: implications for connective tissue growth factor. Thromb Res. 2012; 129:801–06. https://doi.org/10.1016/j.thromres.2011.08.009
5. Greenaway J, Lawler J, Moorehead R, Bornstein P, Lamarre J, Petrik J. Thrombospondin-1 inhibits VEGF levels in the ovary directly by binding and internalization via the low density lipoprotein receptor-related protein-1 (LRP-1). J Cell Physiol. 2007; 210:807–18. https://doi.org/10.1002/jcp.20904
6. Tang M, Zhou F, Zhang W, Guo Z, Shang Y, Lu H, Lu R, Zhang Y, Chen Y, Zhong M. The role of thrombospondin-1-mediated TGF-β1 on collagen type III synthesis induced by high glucose. Mol Cell Biochem. 2011; 346:49–56. https://doi.org/10.1007/s11010-010-0590-7
7. George J, Tsutsumi M. siRNA-mediated knockdown of connective tissue growth factor prevents N-nitrosodimethylamine-induced hepatic fibrosis in rats. Gene Ther. 2007; 14:790–803. https://doi.org/10.1038/sj.gt.3302929
8. Zhu Y, Ren W, Sun C, Shi J, Wang Y, Zhang C. Disruption of connective tissue growth factor by short hairpin RNA inhibits collagen synthesis and extracellular matrix secretion in hepatic stellate cells. Liver Int. 2008; 28:632–9. https://doi.org/10.1111/j.1478-3231.2008.01730.x
9. Azad N, Iyer AK, Wang L, Liu Y, Lu Y, Rojanasakul Y. Reactive oxygen species-mediated p38 MAPK regulates carbon nanotube-induced fibrogenic and angiogenic responses. Nanotoxicology. 2013; 7:157–68. https://doi.org/10.3109/17435390.2011.647929
10. Lee MS, Ghim J, Kim SJ, Yun YS, Yoo SA, Suh PG, Kim WU, Ryu SH. Functional interaction between CTGF and FPRL1 regulates VEGF-A-induced angiogenesis. Cell Signal. 2015; 27:1439–48. https://doi.org/10.1016/j.cellsig.2015.04.001

.
